# Chloroplastic ascorbate modifies plant metabolism and may act as a metabolite signal regardless of oxidative stress

**DOI:** 10.1093/plphys/kiae409

**Published:** 2024-08-06

**Authors:** Dávid Tóth, Roland Tengölics, Fayezeh Aarabi, Anna Karlsson, André Vidal-Meireles, László Kovács, Soujanya Kuntam, Tímea Körmöczi, Alisdair R Fernie, Elton P Hudson, Balázs Papp, Szilvia Z Tóth

**Affiliations:** Laboratory for Molecular Photobioenergetics, HUN-REN Biological Research Centre, Institute of Plant Biology, Temesvári krt. 62, Szeged H-6726, Hungary; Doctoral School of Biology, University of Szeged, Közép fasor 52, Szeged H-6722, Hungary; HCEMM-BRC Metabolic Systems Biology Lab, Temesvári krt. 62, Szeged H-6726, Hungary; Synthetic and Systems Biology Unit, HUN-REN Biological Research Centre, Institute of Biochemistry, Temesvári krt. 62, Szeged H-6726, Hungary; Metabolomics Lab, Core Facilities, HUN-REN Biological Research Centre, Temesvári krt. 62, Szeged H-6726, Hungary; Max Planck Institute of Molecular Plant Physiology, Am Mühlenberg 1, Potsdam-Golm D-14476, Germany; Science for Life Laboratory, School of Engineering Science in Chemistry, Biotechnology and Health, KTH Royal Institute of Technology, PO Box 1031, Solna 171 21, Sweden; Laboratory for Molecular Photobioenergetics, HUN-REN Biological Research Centre, Institute of Plant Biology, Temesvári krt. 62, Szeged H-6726, Hungary; Laboratory for Molecular Photobioenergetics, HUN-REN Biological Research Centre, Institute of Plant Biology, Temesvári krt. 62, Szeged H-6726, Hungary; Laboratory for Molecular Photobioenergetics, HUN-REN Biological Research Centre, Institute of Plant Biology, Temesvári krt. 62, Szeged H-6726, Hungary; HCEMM-BRC Metabolic Systems Biology Lab, Temesvári krt. 62, Szeged H-6726, Hungary; Max Planck Institute of Molecular Plant Physiology, Am Mühlenberg 1, Potsdam-Golm D-14476, Germany; Science for Life Laboratory, School of Engineering Science in Chemistry, Biotechnology and Health, KTH Royal Institute of Technology, PO Box 1031, Solna 171 21, Sweden; HCEMM-BRC Metabolic Systems Biology Lab, Temesvári krt. 62, Szeged H-6726, Hungary; Synthetic and Systems Biology Unit, HUN-REN Biological Research Centre, Institute of Biochemistry, Temesvári krt. 62, Szeged H-6726, Hungary; National Laboratory for Health Security, HUN-REN Biological Research Centre, Temesvári krt. 62, Szeged H-6726, Hungary; Laboratory for Molecular Photobioenergetics, HUN-REN Biological Research Centre, Institute of Plant Biology, Temesvári krt. 62, Szeged H-6726, Hungary

## Abstract

Ascorbate (Asc) is a major plant metabolite that plays crucial roles in various processes, from reactive oxygen scavenging to epigenetic regulation. However, to what extent and how Asc modulates metabolism is largely unknown. We investigated the consequences of chloroplastic and total cellular Asc deficiencies by studying chloroplastic Asc transporter mutant lines lacking PHOSPHATE TRANSPORTER 4; 4 and the Asc-deficient *vtc2-4* mutant of Arabidopsis (*Arabidopsis thaliana*). Under regular growth conditions, both Asc deficiencies caused minor alterations in photosynthesis, with no apparent signs of oxidative damage. In contrast, metabolomics analysis revealed global and largely overlapping alterations in the metabolome profiles of both Asc-deficient mutants, suggesting that chloroplastic Asc modulates plant metabolism. We observed significant alterations in amino acid metabolism, particularly in arginine metabolism, activation of nucleotide salvage pathways, and changes in secondary metabolism. In addition, proteome-wide analysis of thermostability revealed that Asc may interact with enzymes involved in arginine metabolism, the Calvin–Benson cycle, and several photosynthetic electron transport components. Overall, our results suggest that, independent of oxidative stress, chloroplastic Asc modulates the activity of diverse metabolic pathways in vascular plants and may act as an internal metabolite signal.

## Teaser

Chloroplastic ascorbate, beyond its known antioxidant function, interconnects and coordinates diverse metabolic pathways in vascular plants and may act as a metabolite signal.

## Introduction

Ascorbate (Asc) is a multifunctional metabolite essential for a range of cellular processes in plants, including cell division, expansion, cell wall hydroxylation, signal transduction, programmed cell death, biosynthesis of hormones, iron uptake, epigenetic regulation as a cofactor for DNA and histone demethylases in the nucleus, and reactive oxygen species (ROS) scavenging (reviewed by Noctor et al. 2018; Smirnoff 2018; Tóth 2023). It also acts as a key factor in integrating the interaction of ethylene and abscisic acid and in regulating ROS levels (Yu et al. 2019).

Asc controls stomatal movement in photosynthetic tissues (Chen and Gallie 2004). In the Mehler peroxidase reaction, Asc contributes to the detoxification of H_2_O_2_ and the regulation of the redox state of the photosynthetic apparatus (Asada 2006). Within the chloroplast, Asc plays multiple roles (Tóth 2023). It may act as an alternative electron donor to photosystem II (PSII) and contribute to the plant's ability to tolerate heat and light stress (Mano et al. 2004; Tóth et al. 2009, 2011). Asc may also donate electrons to PSII and photosystem I (PSI) in bundle sheath cells with a very low oxygen-evolving capacity (Ivanov et al. 2001, 2007). In vascular plants, Asc acts as a cofactor of violaxanthin de-epoxidase, thereby playing an essential role in the process of nonphotochemical quenching (NPQ) allowing the dissipation of excess energy as heat (Bratt et al. 1995; Müller-Moulé et al. 2002; Saga et al. 2010; Hallin et al. 2016). On the other hand, Asc deficiency does not limit NPQ in *Chlamydomonas reinhardtii*, as it is not required as a cofactor for algal-type violaxanthin de-epoxidase (Li et al. 2016; Vidal-Meireles et al. 2020). We also showed that Asc also contributes to dark-induced senescence by inactivating the oxygen-evolving complex (Podmaniczki et al. 2021).

To fulfill the multiple physiological roles of Asc, vascular plants maintain Asc concentrations at high levels of approximately 20 to 30 mm (Zechmann et al. 2011; Zechmann 2018). Under moderate light intensity and short-day conditions, Asc is seemingly in excess in Arabidopsis (*Arabidopsis thaliana*), since an 80% reduction of Asc content causes no changes in the phenotype and fails to induce oxidative stress (Müller-Moulé et al. 2003, 2004; Lim et al. 2016). On the other hand, under severe environmental stress conditions, Asc may become limited, as shown by an increased oxidative stress tolerance of plants with enhanced Asc regeneration (Wang et al. 2010).

The major route of Asc biosynthesis in plants is the Smirnoff–Wheeler pathway (Wheeler et al. 1998; Smirnoff and Wheeler 2024), in which *Vitamin C Defective 2* (*VTC2)*, encoding GDP-L-galactose phosphorylase (AT4G26850 in Arabidopsis), plays a central regulatory role (Bulley and Laing 2016). The *VTC2* gene has a homolog, *VTC5*, which provides a minor contribution to Asc biosynthesis (Linster et al. 2008). Other mammalian-type biosynthesis pathways have also been suggested in plants (Agius et al. 2003; Wolucka and Van Montagu 2003; Lorence et al. 2004), but the seedling lethality of *vtc2/vtc5* double mutations and metabolite analysis strongly suggest that their contribution to Asc biosynthesis is negligible or nonexistent in Arabidopsis leaves (Dowdle et al. 2007; Lim et al. 2016; Kavkova et al. 2019; Smirnoff and Wheeler 2024).

Most of the enzymatic reactions of Asc biosynthesis occur in the cytosol, except the terminal step, catalyzed by L-galactono-1,4-lactone dehydrogenase, which is localized on the inner mitochondrial membrane. From the mitochondria, Asc is transported to the other cell compartments, necessitating specific transporters, since neither Asc nor its oxidized form, dehydroascorbate, can diffuse through membranes. Until now, two Asc transporters have been characterized in plants. AtDTX25, a member of the multidrug and toxic compound extrusion family in Arabidopsis, is a vacuolar Asc transporter that controls intracellular iron cycling in seedlings (Hoang et al. 2021). AtPHT4;4 (AT4G00370.1), a member of the phosphate transporter 4 family of Arabidopsis, is found in the chloroplast envelope membrane (Guo et al. 2008; Fernie and Tóth 2015; Miyaji et al. 2015). AtPHT4;4 knockout mutants (in the Ler-0 background) exhibited an approximately 30% decrease of chloroplastic Asc content and accordingly a slight impairment of the xanthophyll cycle and a decrease in NPQ at high light (HL) (Miyaji et al. 2015).

Asc has been suggested to modify plant metabolism via distinct mechanisms. It has been suggested to maintain cellular redox homeostasis and participate in ROS signaling upon oxidative stress (Fotopoulos et al. 2006; Foyer et al. 2020). Asc also serves as a chaperone for several 2-oxoglutarate-dependent dioxygenases participating in the biosynthesis of e.g. abscisic acid, gibberellins, and salicylic acid. Thereby, Asc was suggested to affect phytohormone-mediated signaling processes and modify stress tolerance and flowering time (Barth et al. 2006; Xiao et al. 2021). Based on the interconnections between the mitochondrial electron transport chain, Asc biosynthesis, and photosynthesis, Asc was also proposed to take part in inter-organellar communication by acting as a metabolite signal (Rosado-Souza et al. 2020).

Chloroplastic Asc may play a central role in modulating metabolism since the chloroplast is a major sensor of environmental and metabolic changes, with its role in light and temperature acclimation well acknowledged (Kleine et al. 2021; Schwenkert et al. 2022). In addition, chloroplast-to-nucleus retrograde signaling adjusts gene expression and metabolism in order to maintain plant fitness (e.g. Leister 2019).

In order to test the hypothesis that chloroplastic Asc may act as a metabolite signal, we compared the physiological and metabolic consequences of total cellular and chloroplastic Asc deficiencies. To this end, we employed an Asc biosynthesis mutant (*vtc2-4*) and two PHT4;4 mutant lines in Col-0 background (*pht4;4-3* and *pht4;4-4*) to compare overall and chloroplastic deficiencies. Under moderate light intensities, both Asc deficiencies result in barely discernible effects on photosynthesis without signs of oxidative damage. On the other hand, we found widespread changes in the metabolome, involving remarkably similar metabolite sets upon total cellular and chloroplastic Asc deficiencies. Finally, proteome integral solubility alteration (PISA) assay suggests that the modulating effect of Asc is exerted via interactions with specific chloroplastic proteins.

## Results

### Characterization of chloroplastic Asc transporter mutants

**Figure 1.**
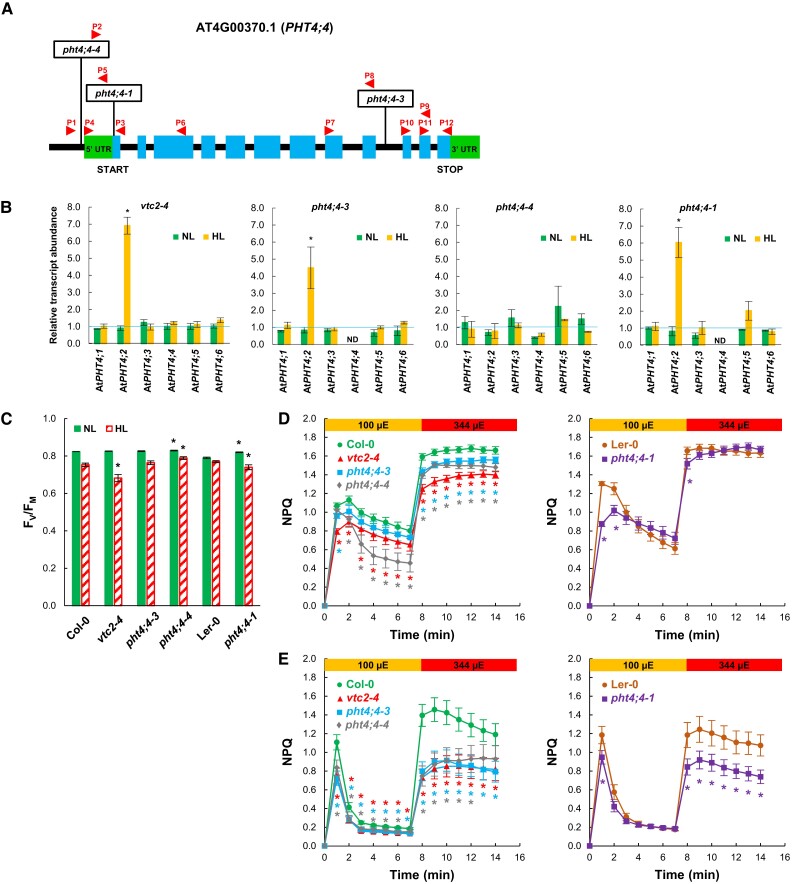
Characterization of *pht4;4* and *vtc2-4* mutants of Arabidopsis. **A)** Physical map of *PHT4;4* (obtained from Phytozome, version 13) with the transposon and T-DNA insertion sites in the *pht4;4* mutants. Exons are shown in blue and 5′UTR and 3′UTR in green. The insertion sites are indicated by boxes, and the binding sites of the primers used for genotyping and gene expression analysis by arrows. **B)** Transcript levels of several *PHT4* genes as determined by RT-qPCR in *vtc2-4*, *pht4;4-3, pht4;4-4,* and *pht4;4* mutants relative to their respective WTs, at NL (approximately 80 *μ*mol photons m^−2^ s^−1^) or at HL (approximately 800 *μ*mol photons m^−2^ s^−1^ for 2 days), grown in three independent experiments. **C)***F*_V_/*F*_M_ values of plants grown at NL (approximately 80 *μ*mol photons m^−2^ s^−1^) or at HL (approximately 800 *μ*mol photons m^−2^ s^−1^ for 2 days). The averages are based on three independent experiments, with nine replicates in each. **D)** Induction of NPQ at 100 and 344 *μ*mol photons m^−2^ s^−1^ of plants grown at NL. The averages are based on three independent experiments, with six biological replicates in each. **E)** Induction of NPQ at 100 and 344 *μ*mol photons m^−2^ s^−1^ of plants grown at HL. The averages are based on three independent experiments, with five replicates in each. In panels **B)** to **E)** averages and standard errors (±SE) are presented. Statistical significance levels between the mutants and their background strains were analyzed using Welch's unpaired *t*-test. The significance levels are presented as *. Primers are listed in Supplementary Table S2.

The available and characterized Asc-deficient *VTC2* mutants are in Col-0 background (such as *vtc2-4*, used in this study and earlier in Lim et al. (2016) and Podmaniczki et al. (2021)), whereas the *pht4;4-1* mutant described by Miyaji et al. (2015) is in Ler-0 background. In order to improve the comparability of overall and chloroplastic Asc deficiencies, we employed PHT4;4 mutants in Col-0 background that we named *pht4;4-3* (called *pht4;4* in Nam et al. (2021)), and *pht4;4-4*. Homozygous T-DNA insertion mutants were identified via PCR, and the exact positions of the T-DNA insertions were determined by sequencing (Fig. 1A). In the *pht4;4-3* mutant, the insertion is found in the ninth intron, whereas in the *pht4;4-4* mutant, it is located in the 5′UTR region.

The *PHT4;4* transcript, as determined by RT-PCR, was absent in the *pht4;4-1* and the *pht4;4-3* mutants, whereas it was moderately expressed in the *pht4;4-4* mutant (Supplementary Fig. S1A). *VTC2* transcript was detected at an equal level in all genotypes, except for the *vtc2-4* mutant, as expected (Supplementary Fig. S1A).

RT-qPCR analysis confirmed that the *PHT4;4* transcript was absent in the *pht4;4-1* and *pht4;4-3* mutants when grown at normal light (NL, 80 *µ*mol photons m^−2^ s^−1^) and kept at HL intensity (800 *µ*mol photons m^−2^ s^−1^) for 2 days. In the *pht4;4-4* mutant, approximately 50% transcript abundance was observed relative to wild type (WT) at both light intensities (Fig. 1B). The transcript levels of other PHT4 family members, which may possibly contribute to chloroplastic Asc transport, were also assessed. *PHT4;1*, *PHT4;3*, *PHT4;5*, and *PHT4;6* were mostly unaltered at both light conditions, whereas at HL, the transcript abundance of *PHT4;2* increased several folds in the *pht4;4* knockout mutants and the *vtc2-4* mutant relative to their respective WT.

### The *vtc2-4* and the *pht4;4* mutants do not exhibit stress symptoms at NL

Next, photosynthetic parameters were compared in plants kept under NL and HL conditions. The *F*_V_/*F*_M_ value, a widely used indicator for PSII photochemistry (Schansker et al. 2014; Stirbet et al. 2018), was similar (about 0.82) in all genotypes at NL, except for Ler-0, which displayed a slightly lower *F*_V_/*F*_M_ value (about 0.79, Fig. 1C). Following HL treatment, the *F*_V_/*F*_M_ value slightly decreased in all genotypes with the most substantial changes in the *vtc2-4* mutant.

In the following experiments, NPQ was assessed upon adaptation to 100 and 344 *µ*mol photons m^−2^ s^−1^ red light. As expected, NPQ was lower in the *vtc2-4* mutant than in Col-0 when grown at NL (in agreement with Müller-Moulé et al. (2002, 2004)) and NPQ was also diminished in the *pht4;4* mutants (Fig. 1D). Upon HL treatment, the diminishment of NPQ was more enhanced in both the *vtc2-4* and the *pht4;4* mutants, especially when determined at 344 *µ*mol photons m^−2^ s^−1^ red light (Fig. 1E).

**Figure 2.**
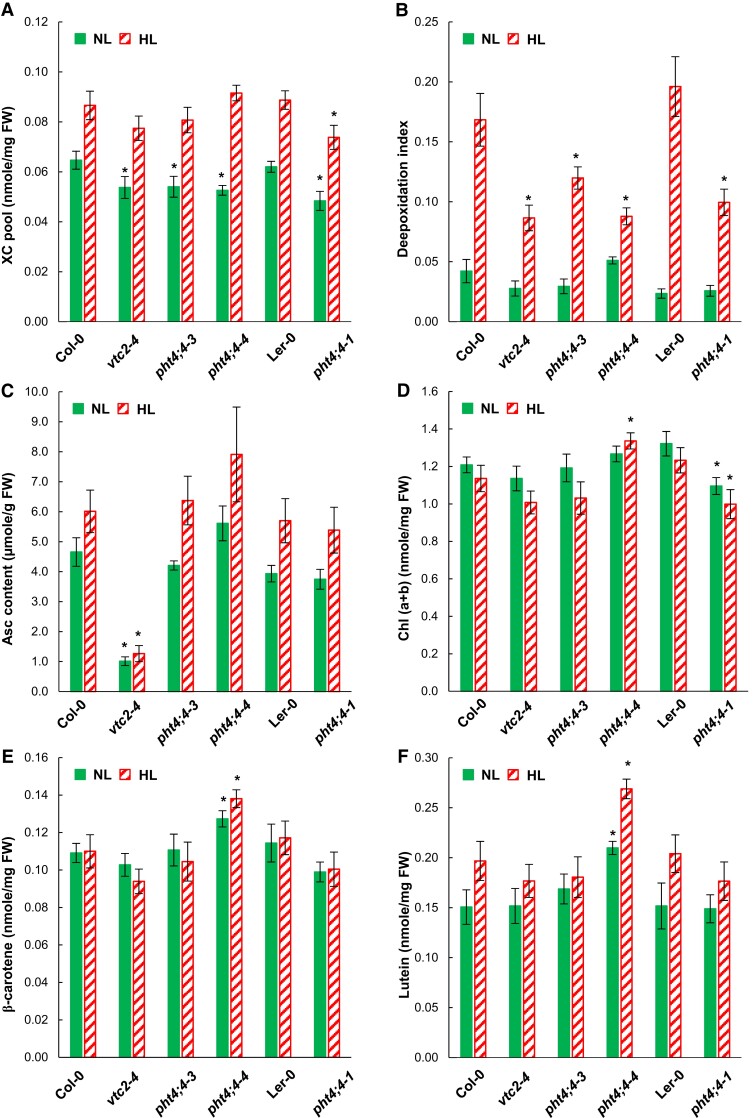
Carotenoids, Asc and Chl(*a* + *b*) contents in *pht4;4-1*, *pht4;4-3*, and *vtc2-4* mutants and their respective WTs (Ler-0 and Col-0) grown at NL (80 *μ*mol photons m^−2^ s^−1^) or at HL (800 *μ*mol photons m^−2^ s^−1^ for 2 days). **A)** Xanthophyll cycle pigment pool size (violaxanthin, antheraxanthin, and zeaxanthin, **B)** de-epoxidation index, **C)** Asc content, **D)** Chl(*a* + *b*) content, **E)**, β-carotene content, and **F)** lutein content on a fresh weight basis. All the averages in panels **A)** to **F)** are based on three independent experiments with nine samples of each treatment and genotype, and standard errors (±SE) are presented. Statistical significance levels between the mutants and their background strains were analyzed using Welch's unpaired *t*-test. The significance levels are presented as * *P* < 0.1.

The xanthophyll cycle pool (violaxanthin, antheraxanthin, and zeaxanthin) was about 20% smaller in the *vtc2-4* and *pht4;4* mutants relative to their WTs at NL. The pool size moderately increased upon the HL treatment in all genotypes (Fig. 2A). The de-epoxidation indices were similar in the mutants and the WTs when grown at NL (Fig. 2B). Upon HL treatment, the de-epoxidation index strongly increased, with augmentation being significantly less substantial in the Asc-deficient *vtc2-4* mutant and all *pht4;4* mutants compared to their respective WTs (Fig. 2B).

As expected, the total Asc content was low in the *vtc2-4* mutant (about 20% of Col-0, Fig. 2C; Lim et al. 2016). The Asc content of the *pht4;4* mutants was similar to their WTs at NL. Upon HL treatment, an approximately 30% to 50% increase was observed in all genotypes, with the exception of the *vtc2-4* mutant (Fig. 2C).

The Chl(*a* + *b*) contents were similar in all genotypes under NL conditions except for *pht4;4-1*, in which the Chl(*a* + *b*) content was significantly lower than in its WT (Fig. 2D). There was a slight decrease in the Chl(*a* + *b*) content upon the HL treatment in all genotypes except the *pht4;4-4* mutant (Fig. 2D). The β-carotene contents were similar in all genotypes both under NL conditions and HL treatment with the exception of the *pht4;4-4* mutant that had a slightly higher β-carotene content (Fig. 2E). The lutein contents of the various genotypes were also very similar under NL conditions, except for *pht4;4-4* in which it was slightly increased relative to Col-0. Upon HL treatment, the lutein content increased slightly (by 7% to 18% in *vtc2-4*, *pht4;4-3*, and *pht4;4-1*) or moderately (by 28% to 34% in Col-0, *pht4;4-4*, and Ler-0; Fig. 2F).

In summary, the *vtc2-4* mutant and the *pht4;4* mutants do not exhibit any stress symptoms under NL conditions. In HL, they have diminished NPQ and de-epoxidation index but only the *vtc2-4* mutant showed a slightly enhanced stress sensitivity.

To assess the effects of Asc deficiency on the plant metabolome, the *vtc2-4* and the *pht4;4-3* knockout mutants and their Col-0 background seemed to be the most suitable. In order to minimize environmental stress-related effects and to study the direct impact of total cellular and chloroplastic Asc deficiencies, the plants were grown under NL conditions showing no phenotypic differences and stress symptoms, as described above.

### Assessment of chloroplastic Asc and total H_2_O_2_ contents

**Figure 3.**
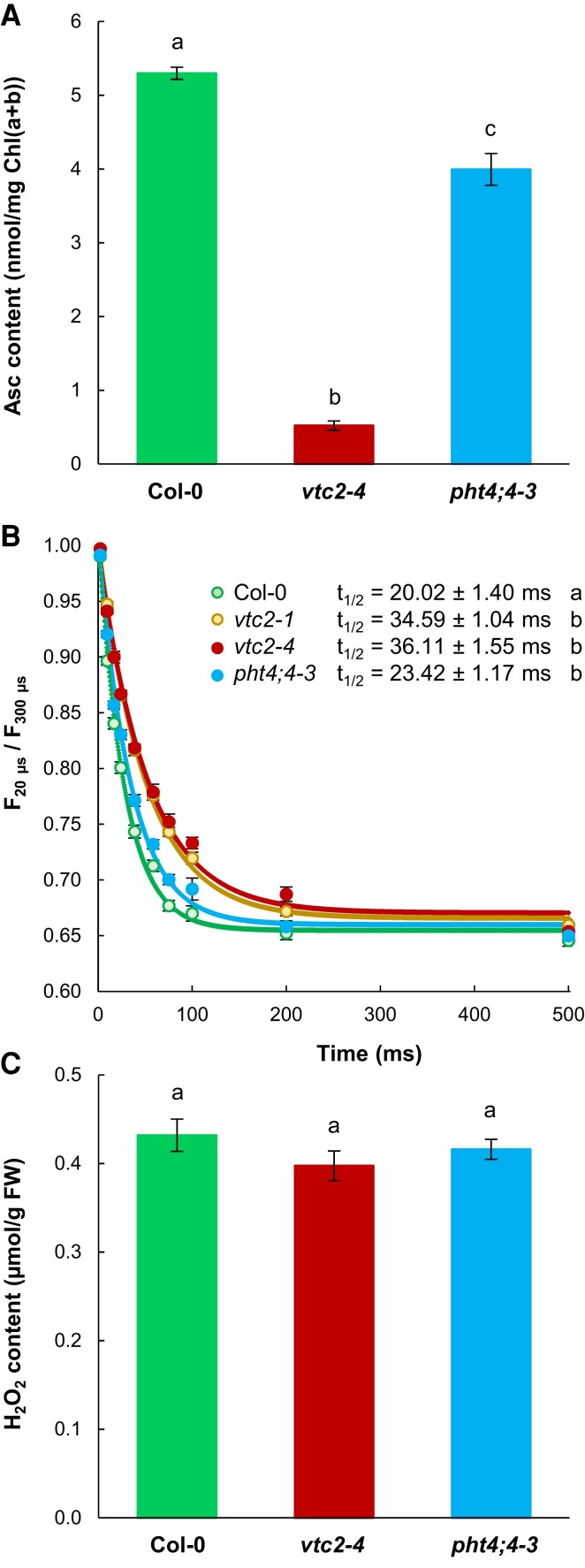
Assessment of chloroplastic Asc and cellular H_2_O_2_ contents. **A)** Asc content of chloroplasts isolated from the *vtc2-4* and *pht4;4-3* mutants and Col-0, quantified by HPLC. The chloroplast intactness was in the range of 80% (see Materials and methods). Data represent averages of three independent biological replicates. **B)** The half-time (t_1/2_) of electron transfer from Asc to PSII in heat-treated (50 °C, 40 s) leaves of *vtc2-4, vtc2-1,* and *pht4;4-3* mutants and Col-0. The t_1/2_ values were derived from the regeneration kinetics of the K-step of the fast Chl *a* fluorescence transient (calculated as *F*_20μs_/*F*_300μs_; see Tóth et al. (2009)). Data represent averages of nine biological replicates. **C)** Total cellular H_2_O_2_ content of *vtc2-4* and *pht4;4-3* mutants and Col-0. Data represent averages of three independent biological replicates. In panels A–C, averages and standard errors (±SE) are presented. The significance of differences between means was determined by ANOVA with Tukey’s post hoc test. Different letters indicate significant differences between means (*P* < 0.1).

To assess the chloroplastic Asc contents of the *vtc2-4* and the *pht4;4-3* mutants and their Col-0 background, we first performed Asc content determinations of isolated chloroplasts having at least 80% integrity (based on Aronsson and Jarvis 2011; Joly and Carpentier 2011). HPLC measurements revealed an approximately 35% decline in chloroplastic Asc level in the *pht4;4-3* mutant and a decrease in the range of 90% for the *vtc2-4* mutant (Fig. 3A).

We have also conducted in vivo Chl *a* fluorescence measurements, triggered by two 5-ms light pulses with varying time intervals, to evaluate the extent of chloroplastic Asc content reduction. This approach leverages the role of Asc as an alternative electron donor to PSII with heat-inactivated oxygen-evolving complexes and has been shown to be sensitive to chloroplastic Asc content (Tóth et al. 2009). The regeneration kinetics of the *F*_20µs_/*F*_300µs_ parameter can be used as an estimate for the half-time (t_1/2_) of electron donation from Asc to PSII, specifically to Tyr_Z_^+^ (Tóth et al. 2009).

Electron donation from Asc to PSII occurred with a t_1/2_ of approximately 20 ms in Col-0. It was slowest in the *vtc2-4* mutant (t_1/2_ approximately 36 ms), similar to the previously reported *vtc2-1* mutant (t_1/2_ approximately 34.6 ms, Fig. 3B). The t_1/2_ in the *pht4;4-3* mutant was approximately 23.4 ms, confirming a milder reduction of chloroplastic Asc content compared to *vtc2-4*.

We have also employed nonaqueous fractionation (NAF) to study the distribution of cellular Asc content between the chloroplast, the cytosol, and the vacuole (Krueger et al. 2011). Results indicate that the chloroplastic Asc level of the *pht4;4-3* mutant is approximately 60% lower than Col-0, while cytosolic Asc is about 100% higher, consistent with its role as an Asc transporter (Supplementary Fig. S2). This method suggests that the *vtc2-4* mutant exhibits a drastic, approximately 95% reduction in chloroplastic Asc content relative to Col-0 (Supplementary Fig. S2).

Thus, based on these three experimental approaches, we conclude an approximately 90% chloroplastic Asc content decrease in the *vtc2-4* mutant and a range of 30% to 50% for the *pht4;4-3 4* mutant compared to Col-0.

Next, we assessed the cellular H_2_O_2_ content. We found that it remained unaltered in the *vtc2-4* and the *pht4;4-3* mutant relative to Col-0 (Fig. 3C), demonstrating that oxidative stress did not occur in these mutants under our growth conditions (short day and 80 *µ*mol photons m^−2^ s^−1^ light intensity). The result is in agreement with Müller-Moulé et al. (2004), who showed that their *vtc2-1* mutant did not express oxidative stress symptoms when grown at moderate light intensity and short-day conditions.

### Untargeted metabolomics reveals a global effect of chloroplastic Asc on the metabolome

Global metabolome analyses were performed on ion intensity data of metabolites annotated at two confidence levels. First, we annotated metabolites based on exact mass only (analogous with level “D” in Alseekh et al. (2021)), yielding 234 putatively annotated metabolites that match compounds in the genome-scale metabolic network reconstruction of Arabidopsis (de Oliveira Dal’Molin et al. 2010). Within these, a subgroup of 118 metabolites could be further identified based on their mass spectrometric fragmentation patterns (analogous to level “B” in Alseekh et al. (2021); Supplementary File 1). The majority (>93%) of the metabolites showed a coefficient of variation (CV) of <40% across biological replicates, indicating high reproducibility.

**Figure 4.**
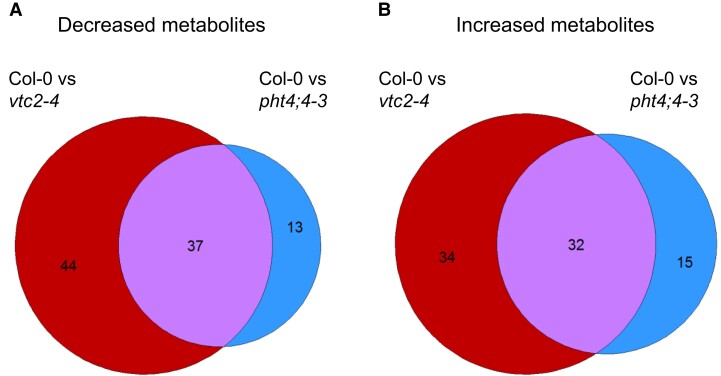
Size-proportional Venn diagrams generated from metabolites annotated at levels “B” and “D” (see Materials and methods). The diagrams represent genotype-specific and overlapping metabolite-level differences that are significantly lower **(A)** or higher **(B)** in both genotypes relative to Col-0. Effect sizes are proportional to circle sizes. On the left (in red) *vtc2-4*-specific changes are shown. On the right (in blue) *pht4;4-3*-specific changes are shown. The intersection range (in purple) represents metabolite-level changes that are significantly changed in both genotypes compared to Col-0.

Principal component analysis (PCA) revealed that the first two components almost entirely separated the three genotypes, and the analysis covered some 48% of the variance (Supplementary Fig. S3), indicating that the metabolome profiles are distinct. Size-proportional Venn diagrams represent genotype-specific and overlapping metabolite-level differences that are significantly altered in the *vtc2-4* and *pht4;4-3* mutants relative to Col-0 (Fig. 4, Supplementary File 1). In the *vtc2-4* mutant, 81 and 66 metabolites were present at a significantly lower and higher level, respectively, relative to Col-0, which is, altogether, about 62% of all metabolites identified at level “D.” A remarkable proportion, approximately 40% of the metabolite changes overlapped between the Asc transporter (*pht4;4-3*) and biosynthesis (*vtc2-4*) mutants.

**Figure 5.**
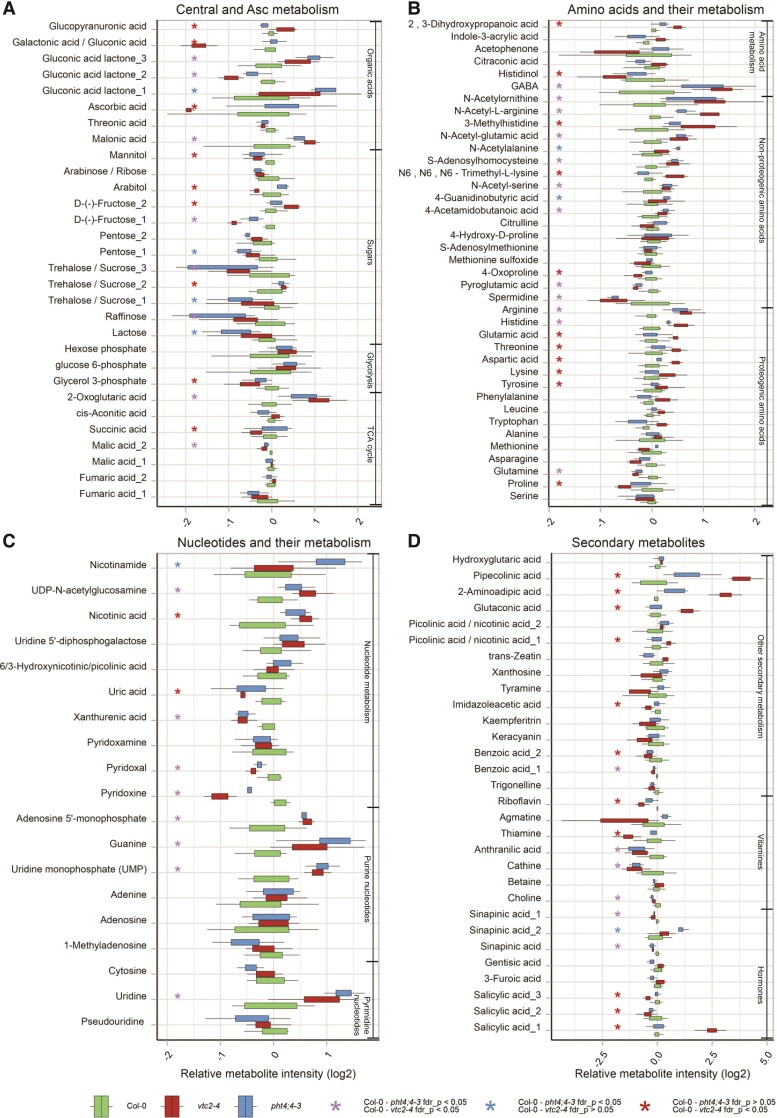
Metabolite levels annotated at level “B” (see Materials and methods), in Col-0, *vtc2-4*, and *pht4;4-3* mutant lines. Box plots show median; interquartile range ± SEM fold change (log2 FC) to average levels in Col-0. Metabolites are grouped into four major compound classes, namely Central and Asc metabolism Central and Asc metabolism **A)**, amino acids and their metabolism **(B)**, nucleotides and their metabolism **(C)**, and secondary metabolism **(D)**. All lines were measured in nine biological replicates in three cultivation batches. Statistics by *t*-test and multiple testing correction using “FDR” were used. Fold changes, *P*-values, and adj. *P*-values are listed in Supplementary File 1. Metabolites are grouped according to their functional classes. Colored “*” indicate the results of pairwise significance tests. Boxes depict the interquartile range (IQR), the solid black lines show the median, and the whiskers extend to 1.5 IQR.

We next analyzed the responses to Asc deficiencies of the 118 individual metabolites annotated at level “B.” Of these metabolites, 67 and 42 were present at significantly different levels from those observed in Col-0 in *vtc2-4* and *pht4;4-3*, respectively (Fig. 5, Supplementary File 1). Of these, 34 metabolites were significantly altered compared to Col-0 in both the *pht4;4-3* and the *vtc2-4* mutants with the same change in direction (i.e. increase or decrease). This overlap is significantly higher compared than what we would expect just by chance based on the number of individual significant differences (randomization test *P* < 10^−5^). Thus, these results show that Asc deficiency induces global metabolome alterations and this effect is largely mediated by chloroplastic Asc content.

Next, Asc deficiencies' effects on the metabolites of the major compound classes were evaluated. A detailed description of the result is given in Supplementary Text S1; here, the major findings are summarized.

Regarding central and amino acid metabolism, we found a significantly increased level of the tricarboxylic acid (TCA) cycle intermediate, 2-oxoglutaric acid in both mutants (Fig. 5A), which may indicate increased respiration (Araújo et al. 2014; Dastogeer et al. 2017). In addition, arginine, a major nitrogen storage compound in the plant cell (reviewed by Siddappa and Marathe 2020), significantly increased in both mutants and glutamate increased in *vtc2-4* (Fig. 5B). Along with this, the level of the plant signaling molecule γ-aminobutyric acid (GABA, Xu et al. 2021) and its derivatives, 4-acetamidobutanoic acid and 4-guaidinobutyric acid, increased slightly in both mutants (Fig. 5B). This may indicate that the GABA shunt was activated to provide alternative carbon sources for mitochondrial respiration, similar to e.g. salt stress (Che-Othman et al. 2020).

We observed that the amounts of proteinogenic amino acids (tyrosine, lysine, aspartic acid, threonine, and glutamic acid) increased mildly in the *vtc2-4* mutant (Fig. 5B), which may result from the activation of their biosynthetic pathways or enhanced protein degradation (Lehmann et al. 2009; Ishikawa et al. 2010; Hildebrandt 2018). The levels of 2-aminoadipic acid and pipecolinic acid, which are both involved in lysine catabolism, have also significantly increased in the *vtc2-4* mutant (Fig. 5D). The levels of these amino acids remained essentially unchanged in the *pht4;4-3* mutant with the exception of histidine that increased in both mutants.

Chloroplastic Asc had a large effect on nucleotide metabolism, too (Fig. 5C). The increase in uridine and uridine monophosphate levels and in malonic acid levels (Fig. 5A) indicates activation of the pyrimidine (uridine) salvage pathway that plays crucial roles in photoassimilate allocation and partitioning (Chen and Thelen 2011). Nicotinic acid levels also increased in both mutants, and the level of nicotinamide was significantly higher in the *pht4;4-3* mutant than in Col-0 (Fig. 5C). These compounds may be re-utilized for the synthesis of pyridine nucleotides by salvage pathways and the synthesis of pyridine alkaloids (Ashihara et al. 2015; Gakière et al. 2018).

We also obtained data on stress-responsive metabolites. The amounts of osmolyte sugars that are known to accumulate under oxidative stress conditions, such as raffinose, fructose, trehalose/sucrose, and mannitol (Baxter et al. 2007; Lehmann et al. 2009), remained unaltered or changed only mildly (Fig. 5A). The amounts of amino acids with antioxidant and osmolyte properties (proline, citrulline, spermidine) also remained unchanged or decreased (Fig. 5B). The amounts of uridine, uridine monophosphate, guanine, and adenosine 5′-monophosphate significantly increased in both mutants (Fig. 5C). Previous studies established that nucleotide metabolism and synthesis are frequently diminished upon oxidative stress (Baxter et al. 2007). Uric acid has been observed to decrease under oxidative stress conditions (Sipari et al. 2020); here, we observed a significant decrease in uric acid level only in the *vtc2-4* mutant (Fig. 5C). The thiamine level slightly decreased in the *vtc2-4*, and it remained at a similar level in the *pht4;4-3* mutant (Fig. 5D). Thiamine functions as an important stress-response molecule that alleviates oxidative stress during different abiotic stress conditions (Tunc-Ozdemir et al. 2009; Rosado-Souza et al. 2020). Similarly, pyridoxine (vitamin B6) and its vitamer derivatives, pyridoxal and pyridoxamine, have been shown to act as antioxidants and their levels increase strongly upon photooxidative stress (Chen and Xiong 2005; Havaux et al. 2009; Parra et al. 2018). Here, we observed a significant decrease in pyridoxine and pyridoxal levels in both mutants (Fig. 5C).

Thus, these results show a global and extensive response of cellular metabolism to the diminishment of total cellular and chloroplastic Asc contents. The most remarkable changes were the activation of the GABA shunt and the upregulation of nucleotide salvage pathways. The considerable overlap between the two *vtc2-4* and the *pht4;4-3* mutants suggests these effects were exerted through chloroplastic Asc content. On the other hand, changes specific to the *vtc2-4* mutant were observed regarding amino acid metabolism. Importantly, no signs of oxidative stress were observed in either mutant, in agreement with the photosynthesis results and the unaltered H_2_O_2_ content (Figs. 1 to 3).

### PISA assay reveals interaction between Asc and chloroplastic proteins

Recent advancements in tandem MS have enabled large-scale analysis of protein structure and conformation. These techniques can determine the thermal denaturation profile of hundreds to thousands of proteins simultaneously, providing insights into protein–protein interactions and other factors influencing protein stability (Volkening et al. 2019).

One approach to elucidate the influence of interacting molecules on protein function involves manipulating extrinsic factors, such as temperature, to induce controlled alterations in protein properties. In thermal proteome profiling (TPP), protein stability is monitored through changes in solubility at different temperatures (Savitski et al. 2014; Mateus et al. 2020). Interaction with certain metabolites can increase or decrease protein stability, either directly through binding or indirectly through affecting protein structure, as demonstrated e.g. for ATP and GTP (Sridharan et al. 2019).

Recently, a high-throughput version of TPP was developed, called PISA assay, which increases the analysis throughput by one to two orders of magnitude (Gaetani et al. 2019). In the PISA assay, a sample is subjected to a treatment expected to induce changes in physicochemical properties and then aliquoted and treated at different temperatures. The increased throughput of the PISA assay compared to regular TPP is achieved through pooling of protein aliquots after individual temperature treatments, reducing the number of analyzed samples. The abundance of the pooled soluble fraction reflects the integral of the melting curve, and comparison with a reference sample allows measurement of relative changes in thermal stability. Unlike conventional TPP, the PISA assay avoids fitting melting curves to the data, mitigating potential bias for proteins with nonstandard melting profiles (Gaetani et al. 2019).

Using the PISA assay, we aimed to test whether Asc interacts with chloroplastic proteins. To this end, we profiled the thermal stability of over 600 chloroplastic Arabidopsis proteins, in the presence of 0, 2, 5, and 10 mm Asc (Supplementary File 1). The employed Asc concentrations are physiologically realistic, as the chloroplastic Asc concentration is in the range of 10 mm in vascular plants (Foyer and Lelandais 1996).

To investigate the stability of Asc during sample preparation for the PISA assay, which involves a 3-min treatment at 40 to 55 °C, we assessed the levels of Asc, dehydroascorbate (DHA), and potential degradation products (2-keto-gulonic acid and L-tartaric acid) using HPLC and MS analyses. Supplementary Figure S4A shows that the Asc content in the chloroplast isolate matched the added 10 mm Asc and remained unchanged following heat treatment at 55 °C. DHA levels were minimal, ranging from 3% to 4% with or without heat treatment. 2-Keto-gulonic acid and L-tartaric acid were found in the μM concentration range, corresponding to a total amount of 0.26% and 0.22% in the room-temperature- and high-temperature-incubated PISA samples, respectively (Supplementary Fig. S4B). These findings suggest that the degradation compounds of Asc are unlikely to affect the PISA assay.

**Figure 6.**
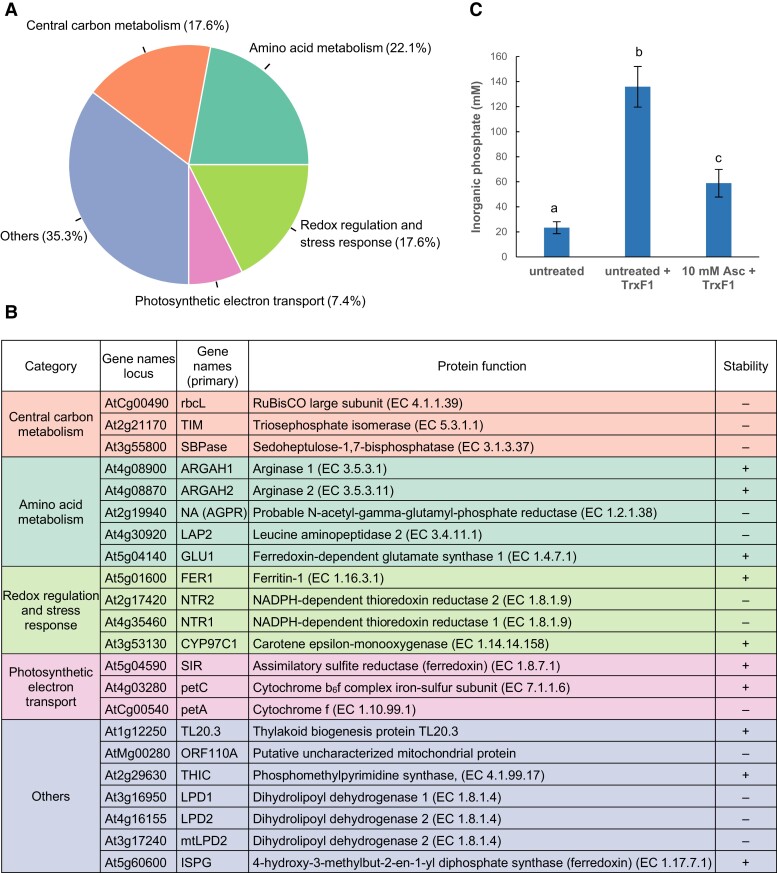
Chloroplastic proteins with altered thermostability (adj. *P*-value <0.05) in response to 5 and 10 mm Asc based on the PISA assay. Proteins were grouped based on the chosen biological processes: photosynthetic electron transport, central carbon metabolism, redox regulation and stress response, and amino acid metabolism. Central carbon metabolism included glycolysis, the pentose phosphate pathway, the tricarboxylic acid cycle, carbon fixation, fatty acid metabolism, and related sugar metabolism. **A)** Classification of major protein groups in response to 10 mm represented as a pie chart **B)** The list of proteins with increased (+) or decreased (−) thermostability in response to 5 mm Asc (see Supplementary File 1). **C)** In vitro activity of SBPase in the presence of 10 mm Asc. SBPase and thioredoxin F1 were expressed in recombinantly in *E. coli* and purified. The assay measures the conversion rate of the alternative SBPase substrate fructose-1-6-bisphosphate to fructose-6-phosphate through the accumulation of inorganic phosphate. Data represent averages of nine biological replicates with standard errors (±SE). The significance of differences between means was determined by ANOVA with Tukey’s post hoc test. The means with different letters are significantly different (*P* < 0.01).

Increased or decreased thermostability for numerous chloroplastic proteins was observed in the presence of 10 mm Asc. At an adjusted (adj.) *P*-value threshold of 0.05, 69 interacting proteins were found, which are involved in central carbon metabolism (17.6%), amino acid metabolism (22.1%), redox regulation and stress response (17.6%), photosynthetic electron transport (7.4%), and other processes or with unknown function (35.3%) (Fig. 6A, Supplementary File 1). Thus, the list of putatively interacting proteins covers a wide range of biological processes, suggesting that Asc has a high potential to modify enzyme activities and/or protein structures.

In the search for high-affinity interactions, we assessed the effects of a lower Asc concentration, 5 mm, on the thermostability of chloroplastic proteins (Fig. 6B). At an adj. *P*-value <0.05, 22 hits were found, of which the majority 14 and 17 were found in the presence of 10 mm Asc at an adj. *P*-value <0.05 and *P*-value <0.1, respectively (Supplementary File 1).

The thermostability of several Calvin–Benson cycle enzymes was diminished by Asc. These include Rubisco large subunit, sedoheptulose-1,7-bisphosphatase (SBPase), and triosephosphate isomerase (Fig. 6B). It has been proposed that the Calvin–Benson cycle enzymes interact within a multienzyme complex (Suss et al. 1993; Graciet et al. 2004; Yu et al. 2020), in which the enzymes are activated under reducing conditions, for instance, by the thioredoxin system (Marri et al. 2009; Kang et al. 2019). We hypothesize that Asc may modify these reactions.

Other possible interacting partners of Asc are arginase 1 (ARGAH1) and arginase 2 (ARGAH2), as shown by increased thermostability in the PISA assay (Fig. 6B). These enzymes are predicted to be localized to the chloroplasts and mitochondria and responsible for the degradation of arginine (Patel et al. 2017; Siddappa and Marathe 2020). The reaction catalyzed by arginase yields ornithine which is the precursor of polyamines, proline, and glutamate that participate in defense against oxidative stress. In addition, the thermostability of At2g19940, which is a putative chloroplast-localized *N*-acetyl-glutamyl-phosphate reductase participating in ornithine biosynthesis (Slocum 2005; Winter et al. 2015), was also altered by Asc (Fig. 6B). In line with these results, the metabolomics analysis (Fig. 5) shows that arginine accumulates, whereas proline, glutathione, and spermidine levels decrease, suggesting that arginase activity was downregulated upon Asc deficiency. In relation to this, the thermostability of leucine aminopeptidase 2 (LAP2) was significantly decreased by Asc. LAP2 liberates N-terminal leucine, methionine, and phenylalanine from proteins and peptides and thereby controls amino acid turnover. Nitrogen-rich amino acids, such as glutamate and glutamine, and GABA levels are particularly affected by LAP2 activity (Waditee-Sirisattha et al. 2011). The effect of Asc on LAP2 thermostability is in line with the changes in GABA content upon Asc deficiency (Fig. 5).

Asc increased the thermostability of ferredoxin-dependent glutamate synthase 1 (GLU) as well, found in both the chloroplast and the mitochondria (Jamai et al. 2009). GLU is responsible for the reassimilation of photorespiratory ammonia as well as for primary nitrogen assimilation in the chloroplast. In this reaction, glutamate is produced from glutamine and 2-oxoglutarate, generated in the TCA cycle (Yoneyama and Suzuki 2020). In broad agreement with the PISA assay, metabolomics showed enhanced glutamate content in the *vtc2-4* Asc-deficient mutant (Fig. 5).

Another potential interacting partner is chloroplastic assimilatory sulfite reductase (SIR, Fig. 6B) that is essential for plant survival due to its role in assimilatory sulfate reduction. Its downregulation causes severe adaptive reactions of primary and secondary metabolism (Khan et al. 2010; Wang et al. 2016). SIR receives electrons from ferredoxin at the acceptor side of PSI, and it reduces sulfite (SO_3_^2−^) to hydrogen sulfide (H_2_S). This step is followed by the incorporation of S_2_^−^ into the amino acid skeleton of the intermediate O-acetylserine (Feldman-Salit et al. 2019) and the generation of cysteine. Besides this reaction, H_2_S also acts as a signaling molecule and thereby modifies plant metabolism at multiple levels (Thakur and Anand 2021). The regulation of SIR is still elusive but must be an integral part of the coordination of the distribution of reducing power from PSI via ferredoxins to the various other consumers (Telman and Dietz 2019).

Asc also affected proteins involved in the regulation of photosynthetic electron transport. We found that thermostability of two subunits of the cytochrome (Cyt) b_6_f complex, namely PetA (Cytf) and PetC (the Rieske iron–sulfur protein), was significantly altered by the Asc treatment (Fig. 6B). The Cytb_6_f complex is known to be particularly suited to sensing the redox state of the electron transport chain and the chloroplast stroma (reviewed by Malone et al. (2021)). In agreement with the PISA assay, it was shown earlier that Asc reduces both PetA and PetC in the low mM range (Riedel et al. 1991; Zhang et al. 2001; Malone et al. 2019). A detailed description of the remaining putative interactions is given in Supplementary Text S2.

Therefore, several lines of evidence suggest that Asc–protein interactions cause at least some of the observed metabolomic changes. We found that the interaction with Asc can lead to both increased and decreased thermal stability of the proteins, as observed, for instance, for ATP as well (Sridharan et al. 2019). The exact nature of each interaction requires further investigations. As a case study, we investigated SBPase, one of the top candidates affected by Asc according to the PISA assay. We recombinantly expressed and purified SBPase in *E. coli*, along with its known activator, thioredoxin F1 (Gütle et al. 2016). Notably, incubating SBPase with 10 mm Asc resulted in a significant decrease (approximately 57%) in its in vitro activity (Fig. 6C). This finding supports the notion that Asc affects SBPase activity, potentially contributing to the observed metabolomic alterations.

## Discussion

It has been hypothesized earlier that chloroplastic Asc may modulate plant metabolism by acting as a metabolite signal (Rosado-Souza et al. 2020). We took two complementary approaches to test this hypothesis. First, we comprehensively compared the physiological and metabolomic effects of total cellular and chloroplastic Asc deficiencies by employing an Asc biosynthesis mutant (*vtc2-4;* Lim et al. 2016) and a knockout mutant of the PHT4;4 transporter (*pht4;4-3*). Second, we used proteome-wide thermostability analysis to reveal potential interactions between Asc and chloroplastic proteins.

The extent of chloroplastic Asc decrease is in the range of 90% in *vtc2* mutants (Fig. 3, Supplementary Fig. S2). Asc is required for violaxanthin de-epoxidase activity (Bratt et al. 1995; Saga et al. 2010; Hallin et al. 2016); accordingly, Asc deficiency diminishes NPQ, particularly in HL (Fig. 1) and the de-epoxidation index was approximately 50% lower in the *vtc2-4* mutant than in Col-0 in HL (Fig. 2B).

In the *pht4;4* mutants, the total cellular Asc concentration is unaltered relative to the WTs (Fig. 2C). We found that the chloroplasts of the *pht4;4-3* mutant contain about 30% to 50% less Asc than WT chloroplasts (Fig. 3, Supplementary Fig. S2). In agreement with this, there was a discernible decrease in NPQ and in the de-epoxidation index in the *pht4;4* mutants relative to their WT (Figs. 1 and 2).

Asc is recognized as one of the most important antioxidants in the cell. In this respect, it is surprising that Asc-deficient mutants have the same levels of β-carotene and lutein as the WT under NL conditions (Fig. 2). The amounts of osmolyte sugars (e.g. raffinose, fructose, mannitol) and amino acids (proline, citrulline, spermidine) participating in oxidative stress response also remained or changed only mildly (Fig. 5), just as well as the cellular H_2_O_2_ content (Fig. 3C). In agreement with our data, it has been observed earlier that under regular short-day conditions, glutathione, Asc peroxidase activity, and the redox state of Asc are also unaltered in the *vtc2* mutants, just as well as photosynthetic activity (Müller-Moulé et al. 2004; Lim et al. 2016), and only moderate changes occur at constant HL (Müller-Moulé et al. 2004). Seedlings of the *pht4;4* mutants also showed no alteration in *F*_V_/*F*_M_, Chl(*a* + *b*), and H_2_O_2_ contents relative to WTs (Miyaji et al. 2015; Nam et al. 2021).

Thus, an approximately 90% reduction of total cellular Asc content in the *vtc2-4* mutant or the 30% to 50% chloroplastic Asc content decrease in the *pht4;4-3* mutant did not lead to photooxidative stress under NL conditions, indicating a margin of safety in Asc levels under such conditions. We note that the partial decrease in chloroplastic Asc content of the *pht4;4-3* mutant suggests that Arabidopsis may possess other, hitherto unknown chloroplastic Asc transporters. A possible candidate may be PHT4;2, whose expression level increased several folds upon HL treatment (Fig. 1); future studies should test this possibility.

To reveal how cellular metabolism is altered upon total cellular and chloroplastic Asc deficiencies, untargeted metabolomics was employed on Col-0, the *vtc2-4* and the *pht4;4-3* knockout mutants. In order to minimize environmental stress-related effects on the metabolome, the plants were grown under precisely controlled NL conditions.

Both total cellular and chloroplastic deficiencies caused large and widespread changes in the metabolome, with the metabolites synthesized in both the chloroplast and the cytosol. The changes affected approximately 57% of the annotated compounds in the *vtc2-4* mutant of which about 51% occurred in the *pht4;4-3* mutant as well (Fig. 5). The fact that the metabolite changes largely overlapped between the mutants suggests that chloroplastic Asc content has a particularly important role in modulating metabolic pathways. In the search for a possible mechanism on how Asc could affect the metabolome, the PISA assay revealed that Asc has a potential to interact with various chloroplastic proteins, participating in arginine metabolism, photosynthetic electron transport, and CO_2_ fixation.

**Figure 7.**
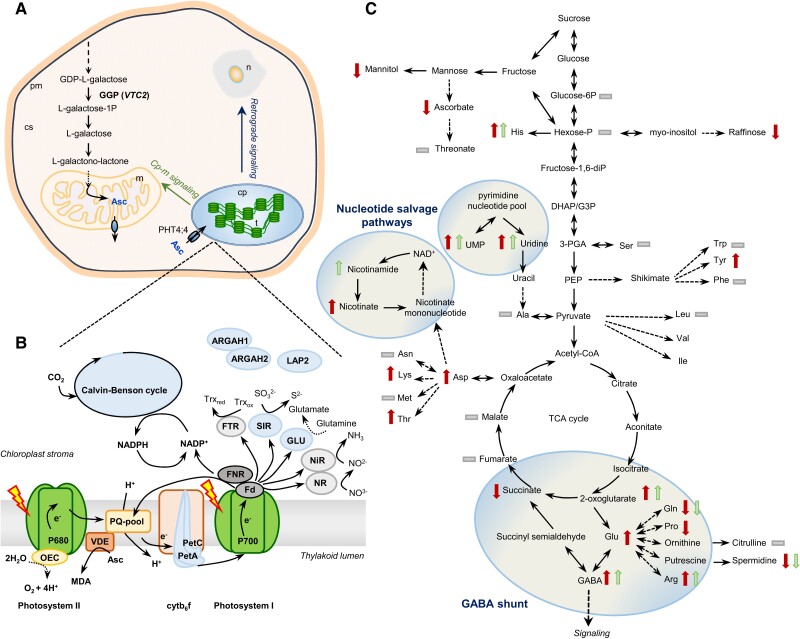
Chloroplastic Asc controls plant metabolism. **A)** Asc biosynthesis takes place in the cytosol, with the exception of the last step, occurring in mitochondria. *vtc2* encodes GDP-L-galactose phosphorylase (GGP), catalyzing the first committed step of Asc biosynthesis. PHT4;4 transports Asc into the chloroplast. **B)** Photosynthetic electron transport components and stromal enzymes potentially interacting with chloroplastic Asc (indicated in blue, based on the PISA assay of this study, see Fig. 6). **C)** Chloroplastic and cellular Asc deficiencies lead to widespread changes in the metabolome, including the induction of the GABA shunt and nucleotide salvage pathways (circled). Dark red and light green arrows indicate significant changes in the respective metabolites identified at level “B” (see Fig. 5) in the *vtc2-4* and the *pht4;4-3* mutants compared to the WT (Col-0), respectively. Gray bars mean that the given metabolite was identified but no significant changes relative to the WT were detected.

Members of the TCA cycle were mildly affected by Asc deficiency, with the exception of 2-oxoglutarate being significantly increased in both mutants. Along with this, the levels of GABA, its derivatives, and arginine significantly increased in both mutants, and glutamate increased in the *vtc2-4* mutant (see Fig. 7), suggesting that the GABA shunt is activated upon chloroplastic Asc deficiency. In line with the increase in arginine content, we found putative interactions between Asc and ARGAH1/ARGAH2, participating in arginine degradation (Patel et al. 2017; Siddappa and Marathe 2020). In relation to this, the thermal stability of LAP2, controlling amino acid turnover, glutamate, glutamine, and GABA levels (Waditee-Sirisattha et al. 2011), was affected by Asc. In addition, the PISA assay also revealed a possible interaction between Asc and GLU, producing glutamate from glutamine and 2-oxoglutarate in the chloroplast (Yoneyama and Suzuki 2020). Thus, Asc may interact with several enzymes regulating the GABA shunt. The main role of the GABA shunt is to provide alternative carbon sources for mitochondrial respiration. GABA has also been described to participate in signal transduction and modulate metabolism in various ways (Xu et al. 2021); therefore, it may potentially transmit the effects of Asc deficiency to various other metabolic pathways. We noted that despite the increase in Glu levels, proline and the polyamine spermidine decreased in both mutants (see Fig. 7).

Cellular and chloroplastic Asc deficiencies also resulted in the induction of nucleotide salvage pathways, probably to fine-tune photoassimilate allocation and partitioning. We note that induction of the GABA shunt along with nicotinamide and uridine salvage pathways was observed earlier upon aging (Schippers et al. 2008; Sipari et al. 2020).

We found that Asc may also affect SIR activity; SIR is another important regulatory enzyme participating in adaptive reactions of primary and secondary metabolism and signaling (Thakur and Anand 2021).

The PISA assay revealed that Asc has the potential to interact with Calvin–Benson cycle enzymes as well, including SBPase. Using in vitro assays, we demonstrated Asc's inhibitory effect on SBPase activity (Fig. 6C). This may be particularly interesting, considering that SBPase activity is associated with growth, starch accumulation, and chilling stress tolerance (Liu et al. 2012; Ding et al. 2017). We note that metabolite-level regulation of the Calvin–Benson cycle activity has been also described in cyanobacteria (Sporre et al. (2023).

Finally, Asc was found to interact with two cytb_6_f components, PetA and PetB. A direct effect of this interaction on the metabolome could not be identified in this study. Nevertheless, we note that the activity of cytb_6_f controls the redox state of the photosynthetic electron transport (Schöttler and Tóth 2014), which is a major retrograde signal, affecting the expression of a large number of genes (Gjindali and Johnson 2023). Therefore, by interacting with PetA and PetB, Asc may participate in retrograde signaling, thereby modulating plant metabolism.

Thus, our results support the earlier hypothesis by Rosado-Souza et al. (2020) and Kleine et al. (2021) that Asc may eventually act as a metabolite signal to mediate metabolic crosstalk between the chloroplast and mitochondria and possibly as a retrograde signal to modify the expression of nuclear genes. As sizeable oxidative stress did not occur, the observed modulating effect of Asc is likely to be unrelated to ROS signaling pathways, although we do not exclude the possibility that under severe stress conditions, the ROS signaling pathways would also be affected by Asc deficiency (Foyer et al. 2020).

A subset of metabolites changed significantly in the *vtc2-4* mutant only (Supplementary Table S1), indicating that these changes are linked to cytosolic Asc content. These include free amino acids, such as tyrosine, lysine, aspartic acid, threonine, and glutamic acid, and secondary metabolites, among which salicylic acid and thiamine have been shown earlier to interact with Asc metabolism and function (Nazar et al. 2015; Rosado-Souza et al. 2020). The synthesis of salicylic acid and thiamine takes place in the cytosol. Thus, it can be assumed that their altered biosynthesis is a consequence of the diminished cytosolic Asc content and/or Asc biosynthesis intermediates. On the other hand, it is also to be considered that in the *pht4;4-3* mutant the reduction in chloroplastic Asc concentration is lower than in the *vtc2-4* mutant (Fig. 3); thus, the effect on the alteration on metabolites may be milder.

As discussed above, substantial oxidative stress was unlikely to occur in the mutants under NL conditions. Therefore, the differences in metabolite levels are not due to oxidative stress but may reflect a specific response to decreased chloroplastic Asc contents. In nature, chloroplastic Asc deficiency may occur under low light conditions, since Asc biosynthesis is known to be strongly dependent on light and the photosynthetic electron transport in particular (Yabuta et al. 2007; Bartoli et al. 2009). Low photosynthetic rate occurs under various stress conditions, namely low temperature (Crosatti et al. 2013), excessive illumination (Gururani et al. 2015), drought stress (Feller 2016), and heat stress (Yamamoto 2016) that may all limit the rate of Asc biosynthesis and, at the same time, enhance Asc oxidation. All these conditions require adaptive responses, and our data suggest that a major player in environmental stress adaptation is Asc, acting as a metabolite signal.

Overall, our study revealed that lowering chloroplastic Asc levels triggers extensive changes in plant metabolism, suggesting a previously unidentified regulatory mechanism. This may involve interactions between chloroplastic Asc and proteins governing photosynthesis, cellular regulation, and, potentially, retrograde signaling. These findings raise the intriguing possibility that plants utilize chloroplastic Asc as a sensor to perceive environmental challenges and adapt their metabolism accordingly.

## Materials and methods

### Plant material and growth conditions

The Asc-deficient *vtc2-4* mutant in the Col-0 background (TAIR stock no. CS69540; see Lim et al. (2016)) originates from the laboratory of Dr. John F. Golz (University of Melbourne, Australia). The *pht4;4-1* mutant (with Ler-0 background; Miyaji et al. 2015) was purchased from TAIR (stock no. N26443). Two AtPHT4;4 mutants in Col-0 background were employed (TAIR stock nos. N469134 (Nam et al. 2021) and N444342), which we named *pht4;4-3* and *pht4;4-4.*

All Arabidopsis (*A. thaliana*) genotypes were initially grown in a growth chamber under short-day conditions (8 h light, 16 h dark), at approximately 80 *µ*mol photons m^−2^ s^−1^ in the light period (normal light, NL) for 8 wk. The temperature was 18 °C in the dark and 22 °C in the light. When the plants were 8 wk old, a subset was transferred to HL (approximately 800 *µ*mol photons m^−2^ s^−1^) for 2 days.

We noted that the Asc-deficient T-DNA insertional mutant *vtc2-4* does not show any alteration in the phenotype in comparison with Col-0 when grown under short-day conditions (in agreement with Lim et al. (2016)). The phenotype of the *pht4;4-3* and *pht4;4-4* mutants was also indistinguishable from their Col-0 background. On the other hand, the *pht4;4-1* mutant plants had long petioles relative to Ler-0, a difference that was not observed by Miyaji et al. (2015) when studying seedlings grown under long-day conditions (Supplementary Fig. S1, B and C).

### PCR, RT-PCR, and RT-qPCR

DNA was isolated based on the protocol of Edwards et al. (1991), and the T-DNA insertion in the *pht4;4-3* and *pht4;4-4* mutants was confirmed by PCR using gene-specific forward, reverse, and T-DNA-specific primers, respectively (see Supplementary Table S2 for the list of primers).

For RNA isolation, approximately 50 to 100 mg of leaf material was collected and frozen in liquid nitrogen before grinding with a MM400 laboratory mill (Retsch, Germany) at 30 Hz for 1 min. Direct-Zol RNA isolation kit was used, following the recommendation of the manufacturer (Zymo Research). RNA integrity was checked on a 1% (w/v) denaturing agarose gel. To remove contaminating DNA from the samples, the isolated RNA was treated with DNaseI (Zymo Research). 1 *µ*g of total RNA was used for cDNA synthesis with random hexamers using FIRESript reverse transcriptase, following the recommendation of the manufacturer (Solis BioDyne). To confirm the absence of transcript in the mutant lines, RT-PCR assays were conducted, consisting of 30 amplification cycles using primers listed in Supplementary Table S2.

The relative transcript abundance of several *PHT4* genes was determined via RT-qPCR following the method described in Vidal-Meireles et al. (2017). The RT-qPCR data are presented as the fold change in mRNA transcript abundance normalized to the average of three reference genes (*GAPDH, ACT2, UBC21*) and relative to the respective WT strains (see Supplementary Table S2 for the list of primers).

### Determination of Asc, chlorophyll, carotenoid, and hydrogen peroxide (H_2_O_2_) content

Total Asc contents of leaves (20 to 40 mg of each sample) and Asc and DHA contents of intact chloroplasts were determined by HPLC, based on the protocol of Kovács et al. (2016).

The assessment of chloroplastic Asc content was performed by NAF. For this, Arabidopsis leaves were harvested and snap-frozen in liquid nitrogen. Two grams of fresh weight of frozen plant material was ground to a fine powder and freeze-dried at 0.02 bar for 5 days in a lyophilizer, which had been pre-cooled to −40 °C. The NAF fractionation procedure was performed as described in Medeiros et al. (2019) and Shapiguzov et al. (2019). Subcellular compartmentation of markers and the metabolites of our interest was calculated by the BestFit method as described in Klie et al. (2011) and Krueger et al. (2011, 2014). BestFit applied the linear regressions for subcellular compartments using the percentage of distribution of each metabolite of our interest and the subcellular markers of each fraction. Marker measurements for NAF were taken as described in Medeiros et al. (2019) and Shapiguzov et al. (2019).

For carotenoid and Chl(*a* + *b*) determination, approximately 10 to 20 mg of Arabidopsis leaves was frozen in liquid nitrogen and ground into a fine powder using an MM400 laboratory mill (Retsch, Germany) at freezing temperature and resuspended in 1 mL acetone. Pigments were extracted for 30 min with continuous shaking at 1,000 rpm at 20 °C in the dark. The extract was centrifuged at 11,500 *g* for 10 min at 4 °C, and the supernatant was collected and passed through a PTFE 0.2-*μ*m pore size syringe filter.

Quantification of carotenoids and Chl(*a* + *b*) was performed by HPLC using a Shimadzu Prominence HPLC system (Shimadzu, Kyoto, Japan) consisting of an LC-20AD pump, a DGU-20A degasser, a SIL-20AC automatic sample injector, a CTO-20AC column thermostat, and a Nexera X2 SPD-M30A photodiode array detector. Chromatographic separations were carried out on a Phenomenex Synergi 4-*µ*m Hydro-RP 80Å, 250 × 4.6 mm column. 20 *μ*L aliquots of acetonic extract was injected into the column, and the pigments were eluted by a linear gradient from solvent A (acetonitrile, water, triethylamine, in a ratio of 9:1:0.01) to solvent B (ethyl acetate) followed by 15-min re-equilibration in solvent A. The gradient from solvent A to solvent B was run from 0 to 25 min at a flow rate of 1 mL/min. The column temperature was set to 25 °C. Eluates were monitored in a wavelength range of 260 to 750 nm. Pigments were identified according to their retention time and absorption spectrum and quantified by integrated chromatographic peak area recorded at the wavelength of maximum absorbance for each kind of pigments using the corresponding molar decadic absorption coefficient (Jeffrey et al. 1997). The de-epoxidation index of the xanthophyll cycle components was calculated as: (zeaxanthin + ½ antheraxanthin)/(violaxanthin + anteraxanthin + zeaxanthin).

Hydrogen peroxide (H_2_O_2_) content in the leaves was determined according to Zsigmond et al. (2024). Briefly, approximately 50 mg of Arabidopsis leaf was harvested, ground in liquid nitrogen, and resuspended in 20 mm potassium phosphate buffer (pH 6.5). The homogenate was centrifuged, and the supernatant was further filtered using an EZ-10 spin column (Bio Basic) to remove cell debris. The filtrate was used to measure the H_2_O_2_ content with the Amplex Red Hydrogen Peroxide/Peroxidase Assay Kit (Thermo Fischer Scientific, Invitrogen), according to the manufacturer’s guidelines. The resulting resorufin production was measured at 560 nm with the Multiscan Go Microplate Spectrophotometer (Thermo Scientific).

### Determination of NPQ and the rate of electron donation from Asc to PSII

NPQ was determined using a Dual-PAM-100 instrument (Heinz Walz GmbH, Germany, Schreiber and Klughammer 2008), on leaves dark-adapted for 30 min. In the initial 7 min, the actinic light intensity was 100 *µ*mol photons m^−2^ s^−1^, and then, the actinic light intensity was increased to 344 *µ*mol photons m^−2^ s^−1^ for seven additional minutes. Saturating pulses of 5000 *µ*mol photons m^−2^ s^−1^ were provided once every minute.

Fast Chl *a* fluorescence measurements were taken at room temperature using a special version of the Handy-PEA instrument (Hansatech Instruments Ltd.) that allows for a reduction in measurement length to 5 ms. Leaf samples were heat-treated in a water bath (50 °C, 40 s) to eliminate the oxygen-evolving activity, enabling the measurement of the rate of electron donation from Asc to PSII (Tóth et al. 2009). Following 15 min of dark adaptation, leaves were illuminated with continuous red light emitted by three LEDs (3,500 *µ*mol photons m^−2^ s^−1^, 650 nm peak wavelength). The first reliably measured point of the fluorescence transient, taken as F_0_, occurred at 20 µs. The measurement length was 5 ms, and the dark intervals between the first and second light pulses were 2.3, 9.6, 16.9, 31.5, 38.8, 53.4, 75.3, 100, 200, or 500 ms. The regeneration kinetics of the K-step, corresponding to the rate of electron transfer from Asc to PSII, were calculated as *F*_20µs_/*F*_300µs_, and the t_1/2_ values were plotted (as described in Tóth et al. 2009).

### Statistics for the physiological and molecular biology experiments

The presented data are based on at least three independent experiments. When applicable, averages and standard errors (±SE) were calculated. Statistical significance levels between the mutants and their background strains under each growth conditions were analyzed using Welch's unpaired *t*-test. The significance levels are presented when applicable as * *P* < 0.1.

### LC/MS analysis

For LC/MS analysis, leaf material was harvested from 8-wk-old Col-0 and *pht4;4-3* mutant plants six hours into the photoperiod. Approximately 50 to 100 mg leaf material was collected, weighted, and frozen in liquid nitrogen before grinding with a MM400 laboratory mill (Retsch, Germany) at 30 Hz for 1 min. After homogenization, the samples were resuspended in extraction buffer (40% [v/v] methanol, 20% [v/v] water, 40% [v/v] acetonitrile). After adding the extraction buffer (1 mL for 80 mg leaves), the samples were vortexed vigorously for 30 s. This was followed by centrifugation at 20,000 *g* at 4 °C for 30 min, and the supernatant was collected. This step was repeated once.

For metabolome analysis, ultrahigh-performance liquid chromatography–tandem mass spectroscopy (UPLC–MS/MS) system, namely a Thermo Q-Exactive Focus instrument (Thermo Fisher Scientific, USA), equipped with a Dionex Unimate 3000 UHPLC system was used. For chromatographic separation, an Xbidge Premier BEH Amide column (2.1 mm × 150 mm, 2.5 *μ*m; Waters Corporation) was used. Each sample was injected twice (2.5 *µ*L), one for the negative ionization mode and another for the positive ionization mode. For negative ionization mode, the mobile phase was gradually changed from 99% (v/v) solvent A (97.5/2.5 acetonitrile/water) to 80% (v/v) solvent B (20/80 acetonitrile/20 mm ammonium acetate + 20 mm ammonia in water) in 14 min using a flow rate of 400 *μ*L/min. In positive ionization mode, the mobile phase was gradually changed from 99% (v/v) solvent A (95/5 acetonitrile/10 mm ammonium formate + 0.125% formic acid in water) to 80% (v/v) solvent B (30/70 acetonitrile/10 mm ammonium formate + 0.125% formic acid in water) in 11 min using a flow rate of 400 *μ*L/min. For acquiring semiquantitative metabolome data, system was operated in MS1 scan mode. Data were acquired using the following settings: sheath gas: 55 L/min, aux gas: 14 L/min, sweep gas: 4 l/min, probe heater 440 °C, and capillary temperature 280 °C with 3,500 V capillary voltage in positive ionization mode and 2,500 V capillary voltage in negative ionization mode. The scanning mass range was 100 to 900 m/z with an agc target 3 × 10^6^ and maximum IT time 250 ms. Pooled extract samples as QCs were injected multiple time across the batch as suggested by the guidelines of Broadhurst et al. (2018).

For metabolite identification, pooled extract samples were injected multiple times at the end of the sequence. For acquiring MS2 data, 10; 30; 50 normalized collision energy (NCE) and 10; 20; 30 collision energy were used for DDMS and PRM modes, respectively, with an automatic ion exclusion time.

### Data processing for metabolomics

To obtain peak intensity values, the recorded data were processed using MZmine2 (Pluskal et al. 2010). Among MZmine modules, rolling ball baseline correction, FTMS peak filtration, ADAP chromatogram deconvolution, ADAP (wavelet) peak detection and peak alignment were used.

Metabolites are reported following recently updated recommendations (Alseekh et al. 2021). Briefly, MS1 level identification (analogous to level “D”) is based on exact mass matching between the given metabolite ion and the metabolites' calculated monoisotopic mass from the Arabidopsis genome-scale metabolic network reconstruction (de Oliveira Dal’Molin et al. 2010). Note that this procedure of metabolite identification is only putative as it may result in multiple equivalently probable metabolite assignments to the same peak, and the same identification may apply to multiple peaks. However, as this procedure is based on an organism-specific metabolic network reconstruction, it is suitable to identify signals originating from Arabidopsis among the 100- and 1,000-s mass spectrometric signals.

MS2 level identification (analogous with level “B” in Alseekh et al. (2021)) is based on MS2 fragmentation spectra. For MS2 level metabolite identification, Compound Discoverer 2.1 was used (similar to e.g. Hemmer et al. 2020) with searching against the Endogenous Metabolites compound class of the mzCloud database (similar to e.g. Klupczynska et al. 2017). Matching scores were accepted above 70, and hits were manually curated. Importantly, this procedure results in high-confidence identifications that are only occasionally ambiguous. All metabolite identification data are reported in Supplementary File 1.

### Data analysis for metabolomics

All statistical procedures were performed using the R statistical language version 4.0. All data are available in the Zenodo repository (https://doi.org/10.5281/zenodo.11354483). Metabolite features were filtered based on detection frequency across multiple injected pooled extract samples (maximum nondetection frequency was set to 12.5%) and peak area reproducibility (CV < 20%). All data were normalized using probabilistic quotient normalization (Dieterle et al. 2006). For PCA, the missing values were input with the KNN method (DMwR2 package). PCA was performed using the prcomp function of stats-package. Statistical analysis and plotting were performed using tidyverse (Wickham et al. 2019) and ggplot (Wickham et al. 2016). Size-proportional Venn diagrams (Euler diagrams) were generated using the Euler function of the eulerr package (Larsson and Gustafsson 2018).

### Chloroplast extraction from Arabidopsis leaves for the PISA assay

Arabidopsis Col-0 plants were grown from seeds at 100 *µ*mol photons s^−1^ m^−2^ in 12-h day/night cycles for 5 wk before harvest. Chloroplasts were isolated from leaves following Aronsson and Jarvis (2011) with some modifications. Leaves were cut and placed in ice-cold isolation buffer (0.3 m sorbitol, 5 mm MgCl_2_, 5 mm EGTA, 5 mm EDTA, 10 mm NaHCO_3_, 20 mm HEPES–KOH pH 8). Harvested leaves were homogenized with an IKA Ultra-Turrax T-25 disperser at 50% power for 3 s and then filtered through double-layered Miracloth. Retentate was resuspended in isolation buffer, homogenized, and filtered. Homogenization was repeated a total of five times. The filtered homogenate was centrifuged at 1,000 g for 5 min at 4 °C, and the pellet was gently resuspended in isolation buffer. A Percoll gradient was prepared by centrifuging equal parts of Percoll and isolation buffer with 0.6 mm glutathione at 43,000 *g* for 30 min at 4 °C, which was subsequently used to separate intact and broken chloroplasts at 7,800 *g* for 10 min at 4 °C. Intact chloroplasts were recovered from the bottom layer, diluted in HEPES-MgSO_4_-Sorbitol (HMS) buffer (50 mm HEPES–NaOH pH 8, 3 mm MgSO_4_, 0.3 m sorbitol), and pelleted at 1,000 *g* for 5 min at 4 °C. Chloroplasts were gently resuspended in 100 *µ*L HMS buffer and illuminated with lamps at 400 *µ*mol photons s^−1^ m^−2^ for 5 min before being snap-frozen in liquid nitrogen. Chlorophyll content was measured in 80% acetone at 647 and 664 nm, and chloroplast intactness was assessed through oxygen evolution as previously described (Joly and Carpentier 2011).

### Proteome integral solubility assay

Frozen chloroplast aliquots were thawed on ice, and 0.8% NP-40 Surfact-Amps Detergent Solution (ThermoFisher, cat. no. 13444269) was added. Chloroplasts were lysed with three cycles of bead beating (45 s, 6.5 m/s; FastPrep-24 5G) with cooling on ice between cycles. Lysates were cleared by centrifugation at (21,000 *g*, 5 min, 4 °C), and endogenous metabolites were removed with Zeba Spin Desalting Columns (ThermoFisher, cat. no. 89882). The lysate was then split into sample replicates for PISA (four replicates for each treatment), with each replicate containing 200 *µ*g of protein, and diluted to a concentration of 1.25 *µ*g/µL in lysis buffer (100 mm HEPES pH 8, 3 mm MgCl_2_, 150 mm KCl). Samples were then incubated in quadruplicates for 10 min under the following four conditions at a protein concentration of 1 *µ*g/µL: (i) lysis buffer, (ii) 2.5 mm Asc, (ii) 5 mm Asc, and (iv) 10 mm Asc. Concentrated solutions of Asc were adjusted to pH 8 in lysis buffer before addition to samples. Samples were then split and incubated in a thermocycler at 16 temperature points between 40 and 55 °C for 3 min. After allowing for precipitation at room temperature for 6 min, temperature points for each replicate were pooled and ultracentrifuged at 150,000 *g* for 30 min. The supernatants were then reduced with 8 mm DTT for 45 min followed by alkylation with 17 mm IAA for 30 min in darkness. Proteins were digested overnight (16 h, 600 rpm) with trypsin/Lys-C protease mix (Thermo Scientific, cat. no. A40009) at a 1:50 enzyme/protein ratio. Following digestion, samples were acidified to pH 2 with formic acid and desalted with pipette tips packed with six layers of C18 Empore SPE Disks (Merck, cat. no. 66883-U) and dried in a Speed Vac at 45 °C for 1 h. For TMT labeling of peptides, 12.5 *µ*g of each sample was resuspended in 20 *µ*L 20 mm EPPS buffer pH 8.5 and labeled with 0.1 mg TMTpro 16plex (Thermo Scientific, cat. no. A44521) for 1 h at 600 rpm. Labeling was quenched by addition of hydroxylamine to 0.37% for 15 min before pooling of samples. Labeled peptides were separated into six fractions with Pierce High pH Reversed-Phase Peptide Fractionation Kit (ThermoFisher, cat. no. 84868) and dried in a Speed Vac at 45 °C for 3.5 h. Dried peptides were stored at −20 °C before LC–MS injection.

### LC/MS analysis of TMT-labeled peptides

Peptides were reconstituted in 0.1% formic acid and analyzed on a Q-Exactive HF hybrid quadrupole-Orbitrap mass spectrometer connected to an UltiMate 3000 RSLCnano System with an EASY-Spray ion source. Peptides were loaded on a C18 Acclaim PepMap 100 trap column (75 *μ*m × 2 cm, 3 *μ*m, 100 Å) with a flow rate of 7 *μ*L per min, using 3% acetonitrile, 0.1% formic acid, and 96.9% water as solvent. Separation was performed using a ES802 EASY-Spray PepMap RSLC C18 Column (75 *μ*m × 25 cm, 2 *μ*m, 100 Å) at a flow rate of 0.7 *μ*L per minute and a 120 min linear gradient from 1% to 32% with 95% acetonitrile, 0.1% formic acid, and 4.9% water as the secondary solvent. Analysis was performed using one full scan (resolution 120,000 at 200 m/z, mass range 350 to 1,500 m/z) followed by 15 MS2 DDA scans with the 15 most abundant peptides (resolution 60,000 at 200 m/z with an isolation window of 0.7 m/z). Precursor ions were fragmented with high-energy collision-induced dissociation at an NCE of 30. Maximum injection time and automatic gain control were set to 50 ms and 3E6 for MS1 and to 120 ms and 1E5 for MS2, respectively.

### Data analysis for the PISA assay

Protein identification and quantification were performed in MaxQuant version 2.2.0.0 using UniProt proteome UP000006548 as a library. Oxidation of methionine and N-terminal acetylation were set as variable modifications, and carbamidomethylation of cysteine was set as a fixed modification. A maximum of two missed cleavages were allowed, and the false discovery rate was set to 1%. MSstats package version 4.4.1 (Choi et al. 2014) was used to median normalize proteins and calculate fold changes in R version 4.2.2. *P*-values were adjusted for multiple hypothesis testing (Benjamini–Hochberg method), and a significance threshold of 0.05 was implemented. Proteins detected in <2 replicates were excluded. Quality assessment statistics and visualizations were generated using R version 4.2.2 and Tidyverse version 1.3.1 (Wickham et al. 2019). The complete dataset is given in Supplementary File 1. Data have been deposited to the ProteomeXchange Consortium via the PRIDE partner repository (Perez-Riverol et al. 2022) with the dataset identifier PXD052756.

### Targeted quantification of Asc degradation intermediates by LC/MS

We followed an established method (Al Kadhi et al. 2017), with modifications for the quantification of Asc degradation intermediates. PISA samples were diluted 10-fold and filtered with an Amicon Ultra centrifugal filter, 3 kDa MWCO (Merck-UFC500324), and 0.5 *µ*L was injected to LC–MS system. Asc degradation intermediates were separated using a Waters HSS T3 column (1.7 *μ*m, 2.1 mm × 100 mm) on a liquid chromatography (Waters ACQUITY Premier) and tandem MS (Waters TQS-Micro) system. Buffer A was composed of LC–MS grade water (VWR-83645.320) and 0.175% formic acid (Sigma 5.33002), and buffer B was composed of 100% acetonitrile (VWR-83640.320). The gradient elution was performed at a constant flow rate of 0.25 mL/min. Starting conditions were 100% A for 2.3 min. From 2.3 to 3 min, B was increased to 30% with a gradient profile of 10, and kept for 0.5 min before returning to the initial conditions. The column was then equilibrated for another 1.2 min, resulting in a 4.7-min chromatographic run. Compounds were identified by matching retention time (1.13 and 1.24 min for 2-keto-gulonic acid and tartaric acid, respectively) and fragmentation (transition: 193 > 58.9 and 149 > 87) with commercially available standards (89986846-Molekula Group; T400-100g Sigma-Aldrich). For MS/MS acquisition with 0.5 kV capillary voltage, the desolvation gas temperature was 600 °C and desolvation gas flow was 1,000 L/min. Signals for organic acids were then acquired in MRM mode in MassLynx software. Quantification was performed using external calibration in the same sample matrix that was used for PISA assay (Rathod et al. 2020).

### Cloning of SBPase and thioredoxin F1

The SBPase gene (At3g55800) and the thioredoxin F1 gene (At3g02730) without transit peptides from Arabidopsis were codon-optimized for *E. coli* and synthesized as geneblocks by Integrated DNA Technologies. The geneblocks were cloned into pET-28a(+) using Gibson assembly. After verification by sequencing, the plasmids were transformed into *E. coli* BL21 using heat shock.

### Protein expression and purification

The BL21 mutants were cultivated in low-salt LB media at 37 °C, 200 rpm until OD_600_ 0.4, whereafter overexpression was induced using 0.5 mm IPTG overnight at 30 °C. Cells were harvested by centrifugation and stored at −20 °C until further processing. Cell pellets were lysed in B-PERTM Complete Bacterial Protein Extraction Reagent (Thermo Fisher Scientific) according to the manufacturer’s instructions, and cell debris was cleared by centrifugation. The supernatant was filtered through a 0.2-*μ*m syringe filter before purification using an ÄKTA start protein purification system with a HisTrap Fast Flow Cytiva column (1 mL). The column was washed with 15 column volumes of wash buffer (50 mm Tris–HCl, 500 mm NaCl, 20 mm imidazole, pH 8) and eluted with a stepwise gradient of elution buffer (50 mm Tris–HCl, 500 mm NaCl, 300 mm imidazole, pH 8). Fractions containing protein were collected, and the buffer was exchanged to storage buffer (50 mm Tris–HCl, pH 8) using a PD-10 Cytiva desalting column. Purified protein was stored at −80 °C in aliquots.

### SBPase activity assay

A malachite green (MG) assay adapted from a previously published protocol (Vardakou et al. 2014) was used to assess the effect of Asc on purified SBPase enzyme. The assay measures the conversion rate of the alternative SBPase substrate fructose-1-6-bisphosphate to fructose-6-phosphate through the accumulation of inorganic phosphate. Phosphate colorimetric development solution was freshly prepared by mixing 400 *µ*L MG dye stock (1.55 g/L MG oxalate salt, 3 m H_2_SO_4_) with 125 *µ*L ammonium molybdate (60 mm) and 10 *µ*L Tween-20 (11% v/v). The solution was filtered through a 0.2-*µ*m filter and kept dark. Development plates were prepared by mixing 36 *µ*L of development solution with 100 *µ*L of reaction buffer (50 mm Tris–HCl, 15 mm MgCl_2_, 10 mm DTT) in 96-well plates and kept dark. Frozen aliquots of SBPase and thioredoxin F1 were thawed on ice and centrifuged at 25,000 × *g* for 5 min to remove precipitated protein. SBPase enzyme was activated for 1 h at room temperature by incubation in reaction buffer with 20 *µ*M thioredoxin F1. To measure the effect of Asc, 25 *µ*L of activated SBPase solution containing 8.4 ng/µL SBPase was incubated for 15 min with 25 *µ*L of reaction buffer with and without 20 *µ*M Asc in quadruplicates. The reaction was initiated by multipipetting 50 *µ*L of fructose-1-6-bisphosphate to a final concentration of 500 *µ*M, and 20 *µ*L of the mixture was immediately sampled to the prepared development plate. Triplicate phosphate standards of 0, 25, 50, and 100 *µ*M were pipetted (20 *µ*L) to the development plate. To stabilize the color, 7.5 *µ*L of sodium citrate (34% w/v) was added to the development plate and the absorbance was measured at 620 nm after 20 min. The sampling of the enzyme reaction mixture was repeated after 30 min with fresh phosphate standards.

### Accession numbers

Sequence data from this article can be found in the GenBank/EMBL data libraries under accession numbers: *VTC2* (At4g26850) and *PHT4;4* (At4g00370).

## Supplementary Material

kiae409_Supplementary_Data

## Data Availability

Raw metabolite profiling data and the complete dataset of the PISA assay are provided in Supplementary File 1. All metabolomics data are available in the Zenodo repository (https://doi.org/10.5281/zenodo.11354483). The MS proteomics data have been deposited to the ProteomeXchange Consortium via the PRIDE partner repository (https://www.ebi.ac.uk/pride/) with the dataset identifier PXD052756.

## Supplementary data

The following materials are available in the online version of this article.

**Supplementary Figure S1.** Characterization of *PHT4;4* and *VTC2* mutants of Arabidopsis.

**Supplementary Figure S2.** The total Asc content, distributed among the chloroplast, the cytosol, and the vacuole, in the *vtc2-4* and *pht4;4-3* mutants and Col-0, assessed by NAF.

**Supplementary Figure S3.** PCA performed on Col-0, *vtc2-4*, and *pht4;4-3* mutant lines.

**Supplementary Figure S4.** Concentration of Asc and its degradation products in PISA samples.

**Supplementary Table S1.** Metabolite changes specific to the *vtc2-4* mutant.

**Supplementary Table S2.** List of primer pairs used in this study.

**Supplementary Text S1.** Detailed description of metabolite changes in the *vtc2-4* and *pht4;4-3* mutants and Col-0.

**Supplementary Text S2.** Additional putative interactions between Asc and chloroplastic proteins indicated by the PISA assay.

**Supplementary File 1.** Metabolite identifications on levels “D” and “B” and intensity fold-change differences; the complete dataset on the PISA assay at 2, 5, and 10 mm Asc. The file contains an explanatory “readme” note.

## References

[kiae409-B1] Agius F , Gonzalez-Lamothe R, Caballero JL, Munoz-Blanco J, Botella MA, Valpuesta V. Engineering increased vitamin C levels in plants by overexpression of a D-galacturonic acid reductase. Nat Biotechnol. 2003:21(2):177–181. 10.1038/nbt777 12524550

[kiae409-B2] Al Kadhi O , Melchini A, Mithen R, Saha S. Development of a LC-MS/MS method for the simultaneous detection of tricarboxylic acid cycle intermediates in a range of biological matrices. J Anal Methods Chem. 2017:2017:5391832. 10.1155/2017/5391832 29075551 PMC5624170

[kiae409-B3] Alseekh S , Aharoni A, Brotman Y, Contrepois K, D’Auria J, Ewald J, Ewald JC, Fraser PD, Giavalisco P, Hall RD, et al Mass spectrometry-based metabolomics: a guide for annotation, quantification and best reporting practices. Nat Methods. 2021:18(7):747–756. 10.1038/s41592-021-01197-1 34239102 PMC8592384

[kiae409-B4] Araújo WL , Martins AO, Fernie AR, Tohge T. 2-Oxoglutarate: linking TCA cycle function with amino acid, glucosinolate, flavonoid, alkaloid, and gibberellin biosynthesis. Front Plant Sci. 2014:5:552. 10.3389/fpls.2014.00552 25360142 PMC4197682

[kiae409-B5] Aronsson H , Jarvis RP. Rapid isolation of Arabidopsis chloroplasts and their use for in vitro protein import assays. In: Jarvis RP, editor. Chloroplast research in Arabidopsis: methods and protocols, volume I. Totowa, NJ: Humana Press; 2011. p. 281–305. 10.1007/978-1-61779-234-2_17 21822845

[kiae409-B6] Asada K . Production and scavenging of reactive oxygen species in chloroplasts and their functions. Plant Physiol. 2006:141(2):391–396. 10.1104/pp.106.082040 16760493 PMC1475469

[kiae409-B7] Ashihara H , Ludwig IA, Katahira R, Yokota T, Fujimura T, Crozier A. Trigonelline and related nicotinic acid metabolites: occurrence, biosynthesis, taxonomic considerations, and their roles in planta and in human health. Phytochem Rev. 2015:14(5):765–798. 10.1007/s11101-014-9375-z

[kiae409-B8] Barth C , De Tullio M, Conklin PL. The role of ascorbic acid in the control of flowering time and the onset of senescence. J Exp Bot. 2006:57(8):1657–1665. 10.1093/jxb/erj198 16698812

[kiae409-B9] Bartoli CG , Tambussi EA, Diego F, Foyer CH. Control of ascorbic acid synthesis and accumulation and glutathione by the incident light red/far red ratio in *Phaseolus vulgaris* leaves. FEBS Lett. 2009:583(1):118–122. 10.1016/j.febslet.2008.11.034 19059408

[kiae409-B10] Baxter CJ , Redestig H, Schauer N, Repsilber D, Patil KR, Nielsen J, Selbig J, Liu J, Fernie AR, Sweetlove LJ. The metabolic response of heterotrophic Arabidopsis cells to oxidative stress. Plant Physiol. 2007:143(1):312–325. 10.1104/pp.106.090431 17122072 PMC1761969

[kiae409-B11] Bratt C , Arvidsson P, Carlsson M, Akerlund H. Regulation of violaxanthin de-epoxidase activity by pH and ascorbate concentration. Photosynth Res. 1995:45(2):169–175. 10.1007/BF00032588 24301483

[kiae409-B12] Broadhurst D , Goodacre R, Reinke SN. Guidelines and considerations for the use of system suitability and quality control samples in mass spectrometry assays applied in untargeted clinical metabolomic studies. Metabolomics. 2018:14(6):72. 10.1007/s11306-018-1367-3 29805336 PMC5960010

[kiae409-B13] Bulley S , Laing W. The regulation of ascorbate biosynthesis. Curr Opin Plant Biol. 2016:33:15–22. 10.1016/j.pbi.2016.04.010 27179323

[kiae409-B14] Che-Othman MH , Jacoby RP, Millar AH, Taylor NL. Wheat mitochondrial respiration shifts from the tricarboxylic acid cycle to the GABA shunt under salt stress. New Phytol. 2020:225(3):1166–1180. 10.1111/nph.15713 30688365

[kiae409-B15] Chen Z , Gallie DR. The ascorbic acid redox state controls guard cell signaling and stomatal movement. Plant Cell. 2004:16(5):1143–1162. 10.1105/tpc.021584 15084716 PMC423206

[kiae409-B16] Chen M , Thelen JJ. Plastid uridine salvage activity is required for photoassimilate allocation and partitioning in Arabidopsis. Plant Cell. 2011:23(8):2991–3006. 10.1105/tpc.111.085829 21828290 PMC3180806

[kiae409-B17] Chen H , Xiong L. Pyridoxine is required for post-embryonic root development and tolerance to osmotic and oxidative stresses. Plant J. 2005:44(3):396–408. 10.1111/j.1365-313X.2005.02538.x 16236150

[kiae409-B18] Choi M , Chang CY, Clough T, Broudy D, Killeen T, Maclean B, Vitek O. MSstats: an R package for statistical analysis of quantitative mass spectrometry-based proteomic experiments. Bioinformatics. 2014:30:2524–2526. 10.1093/bioinformatics/btu305 24794931

[kiae409-B19] Crosatti C , Rizza F, Badeck FW, Mazzucotelli E, Cattivelli L. Harden the chloroplast to protect the plant. Physiol Plantarum. 2013:147(1):55–63. 10.1111/j.1399-3054.2012.01689.x 22938043

[kiae409-B20] Dastogeer KMG , Li H, Sivasithamparam K, Jones M, Du X, Ren Y, Wylie SJ. Metabolic responses of endophytic *Nicotiana benthamiana* plants experiencing water stress. Environ Exp Bot. 2017:143:59–71. 10.1016/j.envexpbot.2017.08.008

[kiae409-B21] de Oliveira Dal’Molin CG , Quek LE, Palfreyman RW, Brumbley SM, Nielsen LK. AraGEM, a genome-scale reconstruction of the primary metabolic network in Arabidopsis. Plant Physiol. 2010:152(2):579–589. 10.1104/pp.109.148817 20044452 PMC2815881

[kiae409-B22] Dieterle F , Ross A, Schlotterbeck G, Senn H. Probabilistic quotient normalization as robust method to account for dilution of complex biological mixtures. Application in ^1^H NMR metabonomics. Anal Chem. 2006:78(13):4281–4290. 10.1021/ac051632c 16808434

[kiae409-B23] Ding F , Wang M, Zhang S. Overexpression of a Calvin cycle enzyme SBPase improves tolerance to chilling-induced oxidative stress in tomato plants. Sci Hortic. 2017:214:27–33. 10.1016/j.scienta.2016.11.010

[kiae409-B24] Dowdle J , Ishikawa T, Gatzek S, Rolinski S, Smirnoff N. Two genes in *Arabidopsis thaliana* encoding GDP-L-galactose phosphorylase are required for ascorbate biosynthesis and seedling viability. Plant J. 2007:52(4):673–689. 10.1111/j.1365-313X.2007.03266.x 17877701

[kiae409-B25] Edwards K , Johnstone C, Thompson C. A simple and rapid method for the preparation of plant genomic DNA for PCR analysis. Nucleic Acids Res. 1991:19:1349. 10.1093/nar/19.6.1349 2030957 PMC333874

[kiae409-B26] Feldman-Salit A , Veith N, Wirtz M, Hell R, Kummer U. Distribution of control in the sulfur assimilation in *Arabidopsis thaliana* depends on environmental conditions. New Phytol. 2019:222(3):1392–1404. 10.1111/nph.15704 30681147

[kiae409-B27] Feller U . Drought stress and carbon assimilation in a warming climate: reversible and irreversible impacts. J Plant Physiol. 2016:203:84–94. 10.1016/j.jplph.2016.04.002 27083537

[kiae409-B28] Fernie AR , Tóth SZ. Identification of the elusive chloroplast ascorbate transporter extends the substrate specificity of the PHT family. Mol Plant. 2015:8(5):674–676. 10.1016/j.molp.2015.02.006 25700636

[kiae409-B29] Fotopoulos V , Sanmartin M, Kanellis AK. Effect of ascorbate oxidase over-expression on ascorbate recycling gene expression in response to agents imposing oxidative stress. J Exp Bot. 2006:57(14):3933–3943. 10.1093/jxb/erl147 16997902

[kiae409-B30] Foyer CH , Kyndt T, Hancock RD. Vitamin C in plants: novel concepts, new perspectives, and outstanding issues. Antioxid Redox Signal. 2020:32(7):463–485. 10.1089/ars.2019.7819 31701753

[kiae409-B31] Foyer CH , Lelandais MA. A comparison of the relative rates of transport of ascorbate and glucose across the thylakoid, chloroplast and plasmalemma membranes of pea leaf mesophyll cells. J Plant Physiol. 1996:148(3–4):391–398. 10.1016/S0176-1617(96)80271-9

[kiae409-B32] Gaetani M , Sabatier P, Saei AA, Beusch CM, Yang Z, Lundström SL, Zubarev RA. Proteome integral solubility alteration: a high-throughput proteomics assay for target deconvolution. J Proteome Res. 2019:18(11):4027–4037. 10.1021/acs.jproteome.9b00500 31545609

[kiae409-B33] Gakière B , Hao J, de Bont L, Pétriacq P, Nunes-Nesi A, Fernie AR. NAD^+^ biosynthesis and signaling in plants. Crit Rev Plant Sci. 2018:37(4):259–307. 10.1080/07352689.2018.1505591

[kiae409-B34] Gjindali A , Johnson GN. Photosynthetic acclimation to changing environments. Biochem Soc Trans. 2023:51(2):473–486. 10.1042/BST20211245 36892145 PMC10212544

[kiae409-B35] Graciet E , Lebreton S, Gontero B. Emergence of new regulatory mechanisms in the Benson-Calvin pathway via protein-protein interactions: a glyceraldehyde-3-phosphate dehydrogenase/CP12/phosphoribulokinase complex. J Exp Bot. 2004:55(400):1245–1254. 10.1093/jxb/erh107 15047759

[kiae409-B36] Guo B , Jin Y, Wussler C, Blancaflor EB, Motes CM, Versaw WK. Functional analysis of the Arabidopsis PHT4 family of intracellular phosphate transporters. New Phytol. 2008:177(4):889–898. 10.1111/j.1469-8137.2007.02331.x 18086223

[kiae409-B37] Gururani MA , Venkatesh J, Tran LSP. Regulation of photosynthesis during abiotic stress-induced photoinhibition. Mol Plant. 2015:8(9):1304–1320. 10.1016/j.molp.2015.05.005 25997389

[kiae409-B38] Gütle DD , Roret T, Müller SJ, Couturier J, Lemaire SD, Hecker A, Dhalleine T, Buchanan BB, Reski R, Einsle O, et al Chloroplast FBPase and SBPase are thioredoxin-linked enzymes with similar architecture but different evolutionary histories. Proc Natl Acad Sci U S A. 2016:113(24):6779–6784. 10.1073/pnas.1606241113 27226308 PMC4914176

[kiae409-B39] Hallin EI , Guo K, Åkerlund H-E. Functional and structural characterization of domain truncated violaxanthin de-epoxidase. Physiol Plantarum. 2016:157(4):414–421. 10.1111/ppl.12428 26864799

[kiae409-B40] Havaux M , Ksas B, Szewczyk A, Rumeau D, Franck F, Caffarri S, Triantaphylidès C. Vitamin B6 deficient plants display increased sensitivity to high light and photo-oxidative stress. BMC Plant Biol. 2009:9(1):130. 10.1186/1471-2229-9-130 19903353 PMC2777905

[kiae409-B41] Hemmer S , Manier SK, Fischmann S, Westphal F, Wagmann L, Meyer MR. Comparison of three untargeted data processing workflows for evaluating LC-HRMS metabolomics data. Metabolites. 2020:10(9):378. 10.3390/metabo10090378 32967365 PMC7570355

[kiae409-B42] Hildebrandt TM . Synthesis versus degradation: directions of amino acid metabolism during Arabidopsis abiotic stress response. Plant Mol Biol. 2018:98(1–2):121–135. 10.1007/s11103-018-0767-0 30143990

[kiae409-B43] Hoang MTT , Almeida D, Chay S, Alcon C, Corratge-Faillie C, Curie C, Mari S. AtDTX25, a member of the multidrug and toxic compound extrusion family, is a vacuolar ascorbate transporter that controls intracellular iron cycling in Arabidopsis. New Phytol. 2021:231(5):1956–1967. 10.1111/nph.17526 34080200

[kiae409-B44] Ishikawa T , Takahara K, Hirabayashi T, Matsumura H, Fujisawa S, Terauchi R, Uchimiya H, Kawai-Yamada M. Metabolome analysis of response to oxidative stress in rice suspension cells overexpressing cell death suppressor Bax inhibitor-1. Plant Cell Physiol. 2010:51(1):9–20. 10.1093/pcp/pcp162 19919949

[kiae409-B45] Ivanov B , Asada K, Edwards GE. Analysis of donors of electrons to photosystem I and cyclic electron flow by redox kinetics of P700 in chloroplasts of isolated bundle sheath strands of maize. Photosynth Res. 2007:92(1):65–74. 10.1007/s11120-007-9166-0 17551845

[kiae409-B46] Ivanov BN , Sacksteder CA, Kramer DM, Edwards GE. Light-induced ascorbate-dependent electron transport and membrane energization in chloroplasts of bundle sheath cells of the C_4_ plant maize. Arch Biochem Biophys. 2001:385(1):145–153. 10.1006/abbi.2000.2156 11361011

[kiae409-B47] Jamai A , Salomé PA, Schilling SH, Weber APM, McClung CR. Arabidopsis photorespiratory serine hydroxymethyltransferase activity requires the mitochondrial accumulation of ferredoxin-dependent glutamate synthase. Plant Cell. 2009:21(2):595–606. 10.1105/tpc.108.063289 19223513 PMC2660619

[kiae409-B48] Jeffrey SW , Mantoura RFC, Wright SW. Phytoplankton pigments in oceanography: guidelines to modern methods. Paris: UNESCO Publishing; 1997.

[kiae409-B49] Joly D , Carpentier R. Rapid isolation of intact chloroplasts from spinach leaves. In: Carpentier R, editor. Photosynthesis research protocols. Methods in molecular biology, vol 684. Totowa (NJ): Humana Press; 2011. p. 321–325. 10.1007/978-1-60761-925-3_24 20960139

[kiae409-B50] Kang Z , Qin T, Zhao Z. Thioredoxins and thioredoxin reductase in chloroplasts: a review. Gene. 2019:706:32–42. 10.1016/j.gene.2019.04.041 31028868

[kiae409-B51] Kavkova I , Blöchl C, Tenhaken R. The Myo-inositol pathway does not contribute to ascorbic acid synthesis. Plant Biol. 2019:21(S1):95–102. 10.1111/plb.12898 30102814 PMC6492119

[kiae409-B52] Khan MS , Haas FH, Samami AA, Gholami AM, Bauer A, Fellenberg K, Reichelt M, Hänsch R, Mendel RR, Meyer AJ, et al Sulfite reductase defines a newly discovered bottleneck for assimilatory sulfate reduction and is essential for growth and development in *Arabidopsis thaliana*. Plant Cell. 2010:22(4):1216–1231. 10.1105/tpc.110.074088 20424176 PMC2879758

[kiae409-B53] Kleine T , Nägele T, Neuhaus HE, Schmitz-Linneweber C, Fernie AR, Geigenberger P, Grimm B, Kaufmann K, Klipp E, Meurer J, et al Acclimation in plants—the Green Hub consortium. Plant J. 2021:106(1):23–40. 10.1111/tpj.15144 33368770

[kiae409-B54] Klie D , Krueger S, Krall L, Giavalisco P, Flügge U-I, Willmitzer L, Steinhauser D. Analysis of the compartmentalized metabolome—a validation of the non-aqueous fractionation technique. Front Plant Sci. 2011:2:55. 10.3389/fpls.2011.00055 22645541 PMC3355776

[kiae409-B55] Klupczynska A , Dereziński P, Garrett TJ, Rubio VY, Dyszkiewicz W, Kasprzyk M, Kokot ZJ. Study of early stage non-small-cell lung cancer using Orbitrap-based global serum metabolomics. J Cancer Res Clin Oncol. 2017:143(4):649–659. 10.1007/s00432-017-2347-0 28168355 PMC5352735

[kiae409-B56] Kovács L , Vidal-Meireles A, Nagy V, Tóth SZ. Quantitative determination of ascorbate from the green alga *Chlamydomonas reinhardtii* by HPLC. Bio-Protocol. 2016:6(24):e2067. 10.21769/BioProtoc.2067

[kiae409-B57] Krueger S , Giavalisco P, Krall L, Steinhauser M-C, Büssis D, Usadel B, Flügge U-I, Fernie AR, Willmitzer L, Steinhauser D. A topological map of the compartmentalized *Arabidopsis thaliana* leaf metabolome. PLoS One. 2011:6(3):e17806. 10.1371/journal.pone.0017806 21423574 PMC3058050

[kiae409-B58] Krueger S , Steinhauser D, Lisec J, Giavalisco P. Analysis of subcellular metabolite distributions within *Arabidopsis thaliana* leaf tissue: a primer for subcellular metabolomics. Methods Mol Biol. 2014:1062:575–596. 10.1007/978-1-62703-580-4_30 24057387

[kiae409-B59] Larsson J , Gustafsson P. A case study in fitting area-proportional Euler diagrams with ellipses using eulerr. In: *Proceedings of international workshop on set visualization and reasoning*. Vol. 2116. 2018. p. 84-91. https://cran.rproject.org/package=eulerr

[kiae409-B60] Lehmann M , Schwarzländer M, Obata T, Sirikantaramas S, Burow M, Olsen CE, Tohge T, Fricker MD, Møller BL, Fernie AR, et al The metabolic response of Arabidopsis roots to oxidative stress is distinct from that of heterotrophic cells in culture and highlights a complex relationship between the levels of transcripts, metabolites, and flux. Mol Plant. 2009:2(3):390–406. 10.1093/mp/ssn080 19825624

[kiae409-B61] Leister D . Piecing the puzzle together: the central role of reactive oxygen species and redox hubs in chloroplast retrograde signaling. Antiox Redox Signal. 2019:30(9):1206–1219. 10.1089/ars.2017.7392 29092621

[kiae409-B62] Li Z , Peers G, Dent RM, Bai Y, Yang SY, Apel W, Leonelli L, Niyogi KK. Evolution of an atypical de-epoxidase for photoprotection in the green lineage. Nat Plants. 2016:2(10):16140. 10.1038/nplants.2016.140 27618685 PMC5021192

[kiae409-B63] Lim B , Smirnoff N, Cobbett CS, Golz JF. Ascorbate-deficient *vtc2* mutants in Arabidopsis do not exhibit decreased growth. Front Plant Sci. 2016:7:1025. 10.3389/fpls.2016.01025 27468291 PMC4943039

[kiae409-B64] Linster CL , Adler LN, Webb K, Christensen KC, Brenner C, Clarke SG. A second GDP-L-galactose phosphorylase in *Arabidopsis* en route to vitamin C—covalent intermediate and substrate requirements for the conserved reaction. J Biol Chem. 2008:283(27):18483–18492. 10.1074/jbc.M802594200 18463094 PMC2441562

[kiae409-B65] Liu X-L , Yu H-D, Guan Y, Li J-K, Guo F-Q. Carbonylation and loss-of-function analyses of SBPase reveal its metabolic interface role in oxidative stress, carbon assimilation, and multiple aspects of growth and development in Arabidopsis. Mol Plant. 2012:5(5):1082–1099. 10.1093/mp/sss012 22402261

[kiae409-B66] Lorence A , Chevone BI, Mendes P, Nessler CL. *myo*-Inositol oxygenase offers a possible entry point into plant ascorbate biosynthesis. Plant Physiol. 2004:134(3):1200–1205. 10.1104/pp.103.033936 14976233 PMC389944

[kiae409-B67] Malone LA , Proctor MS, Hitchcock A, Hunter CN, Johnson MP. Cytochrome b_6_f—orchestrator of photosynthetic electron transfer. Biochim Biophys Acta Bioenerg. 2021:1862(5):148380. 10.1016/j.bbabio.2021.148380 33460588

[kiae409-B68] Malone LA , Qian P, Mayneord GE, Hitchcock A, Farmer DA, Thompson RF, Swainsbury DJK, Ranson NA, Hunter CN, Johnson MP. Cryo-EM structure of the spinach cytochrome b_6_f complex at 3.6 Å resolution. Nature. 2019:575(7783):535–539. 10.1038/s41586-019-1746-6 31723268 PMC7617996

[kiae409-B69] Mano J , Hideg É, Asada K. Ascorbate in thylakoid lumen functions as an alternative electron donor to photosystem II and photosystem I. Arch Biochem Biophys. 2004:429(1):71–80. 10.1016/j.abb.2004.05.022 15288811

[kiae409-B70] Marri K , Zaffagnini M, Collin V, Issakidis-Bourguet E, Lemaire SD, Pupillo P, Sparla F, Miginiac-Maslow M, Trosta P. Prompt and easy activation by specific thioredoxins of Calvin cycle enzymes of *Arabidopsis thaliana* associated in the GAPDH/CP12/PRK supramolecular complex. Mol Plant. 2009:2(2):259–269. 10.1093/mp/ssn061 19825612

[kiae409-B71] Mateus A , Kurzawa N, Becher I, Sridharan S, Helm D, Stein F, Typas A, Savitski MM. Thermal proteome profiling for interrogating protein interactions. Mol Syst Biol. 2020:16(3):e9232. 10.15252/msb.20199232 32133759 PMC7057112

[kiae409-B72] Medeiros DB , Arrivault S, Alpers J, Fernie AR, Aarabi F. Non-aqueous (NAF) for metabolite analysis in subcellular compartments of Arabidopsis leaf tissues. Bio-Protocol. 2019:9(20):e3399. 10.21769/BioProtoc.3399 33654900 PMC7853959

[kiae409-B73] Miyaji T , Kuromori T, Takeuchi Y, Yamaji N, Yokosho K, Shimazawa A, Sugimoto E, Omote H, Ma JF, Shinozaki K, et al AtPHT4;4 is a chloroplast-localized ascorbate transporter in Arabidopsis. Nat Commun. 2015:6(1):5928. 10.1038/ncomms6928 25557369 PMC4308718

[kiae409-B74] Müller-Moulé P , Conklin PL, Niyogi KK. Ascorbate deficiency can limit violaxanthin de-epoxidase activity in vivo. Plant Physiol. 2002:128(3):970–977. 10.1104/pp.010924 11891252 PMC152209

[kiae409-B75] Müller-Moulé P , Golan T, Niyogi KK. Ascorbate-deficient mutants of Arabidopsis grow in high light despite chronic photooxidative stress. Plant Physiol. 2004:134(3):1163–1172. 10.1104/pp.103.032375 14963245 PMC389940

[kiae409-B76] Müller-Moulé P , Havaux M, Niyogi KK. Zeaxanthin deficiency enhances the high light sensitivity of an ascorbate-deficient mutant of Arabidopsis. Plant Physiol. 2003:133(2):748–760. 10.1104/pp.103.026252 12972657 PMC219049

[kiae409-B77] Nam HI , Shahzad Z, Dorone Y, Clowez S, Zhao K, Bouain N, Lay-Pruitt KS, Cho H, Rhee SY, Rouached H. Interdependent iron and phosphorus availability controls photosynthesis through retrograde signaling. Nat Commun. 2021:12(1):7211. 10.1038/s41467-021-27548-2 34893639 PMC8664907

[kiae409-B78] Nazar R , Umar S, Khan NA. Exogenous salicylic acid improves photosynthesis and growth through increase in ascorbate-glutathione metabolism and S assimilation in mustard under salt stress. Plant Signal Behav. 2015:10(3):e1003751. 10.1080/15592324.2014.1003751 25730495 PMC4622964

[kiae409-B79] Noctor G , Reichheld J-P, Foyer CH. ROS-related redox regulation and signaling in plants. Semin Cell Dev Biol. 2018:80:3–12. 10.1016/j.semcdb.2017.07.013 28733165

[kiae409-B80] Parra M , Stahl S, Hellmann H. Vitamin B6 and its role in cell metabolism and physiology. Cells. 2018:7(7):84. 10.3390/cells7070084 30037155 PMC6071262

[kiae409-B81] Patel J , Ariyaratne M, Ahmed S, Ge L, Phuntumart V, Kalinoski A, Morris PF. Dual functioning of plant arginases provides a third route for putrescine synthesis. Plant Sci. 2017:262:62–73. 10.1016/j.plantsci.2017.05.011 28716421

[kiae409-B82] Perez-Riverol Y , Bai J, Bandla C, Hewapathirana S, García-Seisdedos D, Kamatchinathan S, Kundu D, Prakash A, Frericks-Zipper A, Eisenacher M, et al The PRIDE database resources in 2022: a hub for mass spectrometry-based proteomics evidences. Nucleic Acids Res. 2022:50(D1):D543–D552. 10.1093/nar/gkab1038 34723319 PMC8728295

[kiae409-B83] Pluskal T , Castillo S, Villar-Briones A, Orešič M. MZmine 2: modular framework for processing, visualizing, and analyzing mass spectrometry-based molecular profile data. BMC Bioinform. 2010:11(1):395. 10.1186/1471-2105-11-395 PMC2918584 20650010

[kiae409-B84] Podmaniczki A , Nagy V, Vidal-Meireles A, Tóth D, Patai R, Kovács L, Tóth SZ. Ascorbate inactivates the oxygen-evolving complex in prolonged darkness. Physiol Plantarum. 2021:171(2):232–245. 10.1111/ppl.13278 33215703

[kiae409-B85] Rathod R , Gajera B, Nazir K, Wallenius J, Velagapudi V. Simultaneous measurement of tricarboxylic acid cycle intermediates in different biological matrices using liquid chromatography–tandem mass spectrometry; quantitation and comparison of TCA cycle intermediates in human serum, plasma, Kasumi-1 cell and murine liver tissue. Metabolites. 2020:10:103. 10.3390/metabo10030103 32178322 PMC7143453

[kiae409-B86] Riedel A , Rutherford AW, Hauskall G, Muller A, Nitschke W. Chloroplast Rieske Center. EPR study on its spectral characteristics, relaxation and orientation properties. J Biol Chem. 1991:266(27):17838–17844. 10.1016/S0021-9258(18)55204-2 1655728

[kiae409-B87] Rosado-Souza L , Fernie AR, Aarabi F. Ascorbate and thiamin: metabolic modulators in plant acclimation responses. Plants. 2020:9(1):101. 10.3390/plants9010101 31941157 PMC7020166

[kiae409-B89] Saga G , Giorgetti A, Fufezan C, Giacometti GM, Bassi R, Morosinotto T. Mutation analysis of violaxanthin de-epoxidase identifies substrate-binding sites and residues involved in catalysis. J Biol Chem. 2010:285(31):23763–23770. 10.1074/jbc.M110.115097 20507981 PMC2911307

[kiae409-B90] Savitski MM , Reinhard FB, Franken H, Werner T, Savitski MF, Eberhard D, Martinez Molina D, Jafari R, Dovega RB, Klaeger S, et al Tracking cancer drugs in living cells by thermal profiling of the proteome. Science. 2014:346(6205):1255784. 10.1126/science.1255784 25278616

[kiae409-B91] Schansker G , Tóth SZ, Holzwarth AR, Garab G. Chlorophyll *a* fluorescence: beyond the limits of the Q_A_ model. Photosynth Res. 2014:120(1–2):43–58. 10.1007/s11120-013-9806-5 23456268

[kiae409-B92] Schippers JH , Nunes-Nesi A, Apetrei R, Hille J, Fernie AR, Dijkwel PP. The *Arabidopsis onset of leaf death5* mutation of quinolinate synthase affects nicotinamide adenine dinucleotide biosynthesis and causes early ageing. Plant Cell. 2008:20(10):2909–2925. 10.1105/tpc.107.056341 18978034 PMC2590718

[kiae409-B93] Schöttler MA , Tóth SZ. Photosynthetic complex stoichiometry dynamics in higher plants: environmental acclimation and photosynthetic flux control. Front Plant Sci. 2014:5:188. 10.3389/fpls.2014.00188 24860580 PMC4026699

[kiae409-B94] Schreiber U , Klughammer C. Non-photochemical fluorescence quenching and quantum yields in PSI and PSII: analysis of heat-induced limitations using Maxi-Imaging-PAM and Dual-PAM-100. PAM Appl Notes. 2008:1:15–18. https://www.walz.com/files/downloads/pan/PAN07003.pdf.

[kiae409-B95] Schwenkert S , Fernie AR, Geigenberger P, Leister D, Möhlmann T, Naranjo B, Neuhaus HE. Chloroplasts are key players to cope with light and temperature stress. Trends Plant Sci. 2022:27(6):577–587. 10.1016/j.tplants.2021.12.004 35012879

[kiae409-B96] Shapiguzov A , Vainonen JP, Hunter K, Tossavainen H, Tiwari A, Järvi S, Hellman M, Aarabi F, Alseekh S, Wybouw B, et al Arabidopsis RCD1 coordinates chloroplast and mitochondrial functions through interaction with ANAC transcription factors. eLife. 2019:8:e43284. 10.7554/eLife.43284 30767893 PMC6414205

[kiae409-B97] Siddappa S , Marathe GK. What we know about plant arginases? Plant Physiol Biochem. 2020:156:600–610. 10.1016/j.plaphy.2020.10.002 33069114

[kiae409-B98] Sipari N , Lihavainen J, Shapiguzov A, Kangasjärvi J, Keinänen M. Primary metabolite responses to oxidative stress in early-senescing and paraquat resistant *Arabidopsis thaliana rcd1* (*Radical-Induced Cell Death1*). Front Plant Sci. 2020:11:194. 10.3389/fpls.2020.00194 32180786 PMC7059619

[kiae409-B99] Slocum RD . Genes, enzymes and regulation of arginine biosynthesis in plants. Plant Physiol Biochem. 2005:43(8):729–745. 10.1016/j.plaphy.2005.06.007 16122935

[kiae409-B100] Smirnoff N . Ascorbic acid metabolism and functions: a comparison of plants and mammals. Free Radic Biol Med. 2018:122:116–129. 10.1016/j.freeradbiomed.2018.03.033 29567393 PMC6191929

[kiae409-B101] Smirnoff N , Wheeler GL. The ascorbate biosynthesis pathway in plants is known, but there is a way to go with understanding control and functions. J Exp Bot. 2024:75(9):2604–2630. 10.1093/jxb/erad505 38300237 PMC11066809

[kiae409-B102] Sporre E , Karlsen J, Schriever K, Asplund-Samuelsson J, Janasch M, Strandberg L, Karlsson A, Kotol D, Zeckey L, Piazza I, et al Metabolite interactions in the bacterial Calvin cycle and implications for flux regulation. Commun Biol. 2023(6):947. 10.1038/s42003-023-05318-8 37723200 PMC10507043

[kiae409-B103] Sridharan S , Kurzawa N, Werner T, Günthner I, Helm D, Huber W, Bantscheff M, Savitski MM. Proteome-wide solubility and thermal stability profiling reveals distinct regulatory roles for ATP. Nat Commun. 2019:10(1):1155. 10.1038/s41467-019-09107-y 30858367 PMC6411743

[kiae409-B104] Stirbet A , Lazár D, Kromdijk J, Govindjee A. Chlorophyll *a* fluorescence induction: can just a one-second measurement be used to quantify abiotic stress responses? Photosynthetica. 2018:56(SPECIAL ISSUE):86–104. 10.1007/s11099-018-0770-3

[kiae409-B105] Suss KH , Arkona C, Manteuffel R, Adler K. Calvin cycle multienzyme complexes are bound to chloroplast thylakoid membranes of higher plants in situ. Proc Natl Acad Sci USA. 1993:90(12):5514–5518. 10.1073/pnas.90.12.5514 11607406 PMC46751

[kiae409-B106] Telman W , Dietz K-J. Thiol redox-regulation for efficient adjustment of sulfur metabolism in acclimation to abiotic stress. J Exp Bot. 2019:70(16):4223–4236. 10.1093/jxb/erz118 30868161

[kiae409-B107] Thakur M , Anand A. Hydrogen sulfide: an emerging signaling molecule regulating drought stress response in plants. Physiol Plant. 2021:172(2):1227–1243. 10.1111/ppl.13432 33860955

[kiae409-B108] Tóth SZ . The functions of chloroplastic ascorbate in vascular plants and algae. Int J Mol Sci. 2023:24(3):2537. 10.3390/ijms24032537 36768860 PMC9916717

[kiae409-B109] Tóth SZ , Nagy V, Puthur JT, Kovács L, Garab G. The physiological role of ascorbate as photosystem II electron donor: protection against photoinactivation in heat-stressed leaves. Plant Physiol. 2011:156(1):382–392. 10.1104/pp.110.171918 21357184 PMC3091034

[kiae409-B111] Tóth SZ , Puthur JT, Nagy V, Garab G. Experimental evidence for ascorbate-dependent electron transport in leaves with inactive oxygen-evolving complexes. Plant Physiol. 2009:149(3):1568–1578. 10.1104/pp.108.132621 19144767 PMC2649403

[kiae409-B112] Tunc-Ozdemir M , Miller G, Song L, Kim J, Sodek A, Koussevitzky S, Misra AN, Mittler R, Shintani D. Thiamin confers enhanced tolerance to oxidative stress in Arabidopsis. Plant Physiol. 2009:151(1):421–432. 10.1104/pp.109.140046 19641031 PMC2735988

[kiae409-B113] Vardakou M , Salmon M, Faraldos JA, O'Maille PE. Comparative analysis and validation of the malachite green assay for the high throughput biochemical characterization of terpene synthases. MethodsX. 2014:1:187–196. 10.1016/j.mex.2014.08.007 26150952 PMC4472957

[kiae409-B114] Vidal-Meireles A , Neupert J, Zsigmond L, Rosado-Souza L, Kovács L, Nagy V, Galambos A, Fernie AR, Bock R, Tóth SZ. Regulation of ascorbate biosynthesis in green algae has evolved to enable rapid stress-induced response via the VTC2 gene encoding GDP-L-galactose phosphorylase. New Phytol. 2017:214:668–681. 10.1111/nph.14425 28112386

[kiae409-B115] Vidal-Meireles A , Tóth D, Kovács L, Neupert J, Tóth SZ. Ascorbate deficiency does not limit non-photochemical quenching in *Chlamydomonas reinhardtii*. Plant Physiol. 2020:182(1):597–611. 10.1104/pp.19.00916 31662419 PMC6945847

[kiae409-B116] Volkening JD , Stecker KE, Sussman MR. Proteome-wide analysis of protein thermal stability in the model higher plant *Arabidopsis thaliana*. Mol Cell Proteomics. 2019:18(2):308–319. 10.1074/mcp.RA118.001124 30401684 PMC6356070

[kiae409-B117] Waditee-Sirisattha R , Shibato J, Rakwal R, Sirisattha S, Hattori A, Nakano T, Takabe T, Tsujimoto M. The Arabidopsis aminopeptidase LAP2 regulates plant growth, leaf longevity and stress response. New Phytol. 2011:191(4):958–969. 10.1111/j.1469-8137.2011.03758.x 21569035

[kiae409-B118] Wang M , Jia Y, Xu Z, Xia Z. Impairment of sulfite reductase decreases oxidative stress tolerance in *Arabidopsis thaliana*. Front Plant Sci. 2016:7:1843. 10.3389/fpls.2016.01843 27994615 PMC5133253

[kiae409-B119] Wang Z , Xiao Y, Chen W, Tang K, Zhang L. Increased vitamin C content accompanied by an enhanced recycling pathway confers oxidative stress tolerance in Arabidopsis. J Integr Plant Biol. 2010:52(4):400–409. 10.1111/j.1744-7909.2010.00921.x 20377702

[kiae409-B120] Wheeler GL , Jones MA, Smirnoff N. The biosynthetic pathway of vitamin C in higher plants. Nature. 1998:393(6683):365–369. 10.1038/30728 9620799

[kiae409-B121] Wickham H , Averick M, Bryan J, Chang W, McGowan LDA, François R, Grolemund G, Hayes A, Henry L, Hester J, et al Welcome to the Tidyverse. J Open Source Softw. 2019:4(43):1686. 10.21105/joss.01686

[kiae409-B122] Wickham H , Chang W, Wickham MH. Package ‘ggplot2'. Create elegant data visualisations using the grammar of graphics. Version 2(1) 2016, 1–189. https://ggplot2.tidyverse.org/

[kiae409-B123] Winter G , Todd CD, Trovato M, Forlani G, Funck D. Physiological implications of arginine metabolism in plants. Front Plant Sci. 2015:6:534. 10.3389/fpls.2015.00534 26284079 PMC4520006

[kiae409-B124] Wolucka BA , Van Montagu M. GDP-mannose 3′,5′-epimerase forms GDP-L-gulose, a putative intermediate for the de novo biosynthesis of vitamin C in plants. J Biol Chem. 2003:278(48):47483–47490. 10.1074/jbc.M309135200 12954627

[kiae409-B125] Xiao M , Li Z, Zhu L, Wang J, Zhang B, Zheng F, Zhao B, Zhang H, Wang Y, Zhang Z. The multiple roles of ascorbate in the abiotic stress response of plants: antioxidant, cofactor, and regulator. Front Plant Sci. 2021:12:598173. 10.3389/fpls.2021.598173 33912200 PMC8072462

[kiae409-B126] Xu B , Sai N, Gilliham M. The emerging role of GABA as a transport regulator and physiological signal. Plant Physiol. 2021:187(4):2005–2016. 10.1093/plphys/kiab347 35235673 PMC8644139

[kiae409-B127] Yabuta Y , Mieda T, Rapolu M, Nakamura A, Motoki T, Maruta T, Yoshimura K, Ishikawa T, Shigeoka S. Light regulation of ascorbate biosynthesis is dependent on the photosynthetic electron transport chain but independent of sugars in Arabidopsis. J Exp Bot. 2007:58(10):2661–2671. 10.1093/jxb/erm124 17586607

[kiae409-B128] Yamamoto Y . Quality control of photosystem II: the mechanisms for avoidance and tolerance of light and heat stresses are closely linked to membrane fluidity of the thylakoids. Front Plant Sci. 2016:7:1136. 10.3389/fpls.2016.01136 27532009 PMC4969305

[kiae409-B129] Yoneyama T , Suzuki A. Light-independent nitrogen assimilation in plant leaves: nitrate incorporation into glutamine, glutamate, aspartate, and asparagine traced by 15N. Plants. 2020:9(10):1303. 10.3390/plants9101303 33023108 PMC7600499

[kiae409-B130] Yu Y , Wang J, Li S, Kakan X, Zhou Y, Miao Y, Wang F, Qin H, Huang R. Ascorbic acid integrates the antagonistic modulation of ethylene and abscisic acid in the accumulation of reactive oxygen species. Plant Physiol. 2019:179(4):1861–1875. 10.1104/pp.18.01250 30723177 PMC6446745

[kiae409-B131] Yu A , Xie Y, Pan X, Zhang H, Cao P, Su X, Chang W, Li M. Photosynthetic phosphoribulokinase structures: enzymatic mechanisms and the redox regulation of the Calvin-Benson-Bassham cycle. Plant Cell. 2020:32(5):1556–1573. 10.1105/tpc.19.00642 32102842 PMC7203937

[kiae409-B132] Zechmann B . Compartment-specific importance of ascorbate during environmental stress in plants. Antioxid Redox Signal. 2018:29(15):1488–1501. 10.1089/ars.2017.7232 28699398

[kiae409-B133] Zechmann B , Stumpe M, Mauch F. Immunocytochemical determination of the subcellular distribution of ascorbate in plants. Planta. 2011:233(1):1–12. 10.1007/s00425-010-1275-x 20872269 PMC3015205

[kiae409-B134] Zhang H , Whitelegge JP, Cramer WA. Ferredoxin:NADP oxidoreductase is a subunit of the chloroplast cytochrome b_6_f complex. J Biol Chem. 2001:276(41):38159–38165. 10.1074/jbc.M105454200 11483610

[kiae409-B135] Zsigmond L , Juhász-Erdélyi A, Valkai I, Aleksza D, Rigó G, Kant K, Szepesi A, Fiorani F, Körber N, Kovács L, et al Mitochondrial complex I subunit NDUFS8.2 modulates responses to stresses associated with reduced water availability. Plant Physiol Biochem. 2024:208:108466. 10.1016/j.plaphy.2024.108466 38428158

