# Supplementary material for: Chloroplastic ascorbate modifies plant metabolism and may act as a metabolite signal regardless of oxidative stress
Source: Plant Physiol. 2024 Aug 6;196(2):1691–711. doi: 10.1093/plphys/kiae409 (PMC11444284; doi:10.1093/plphys/kiae409)
Supplement: kiae409_Supplementary_Data [file kiae409_supplementary_data.zip › PP2024RA00397DR1_Supplementary Figures and Tables.pdf]

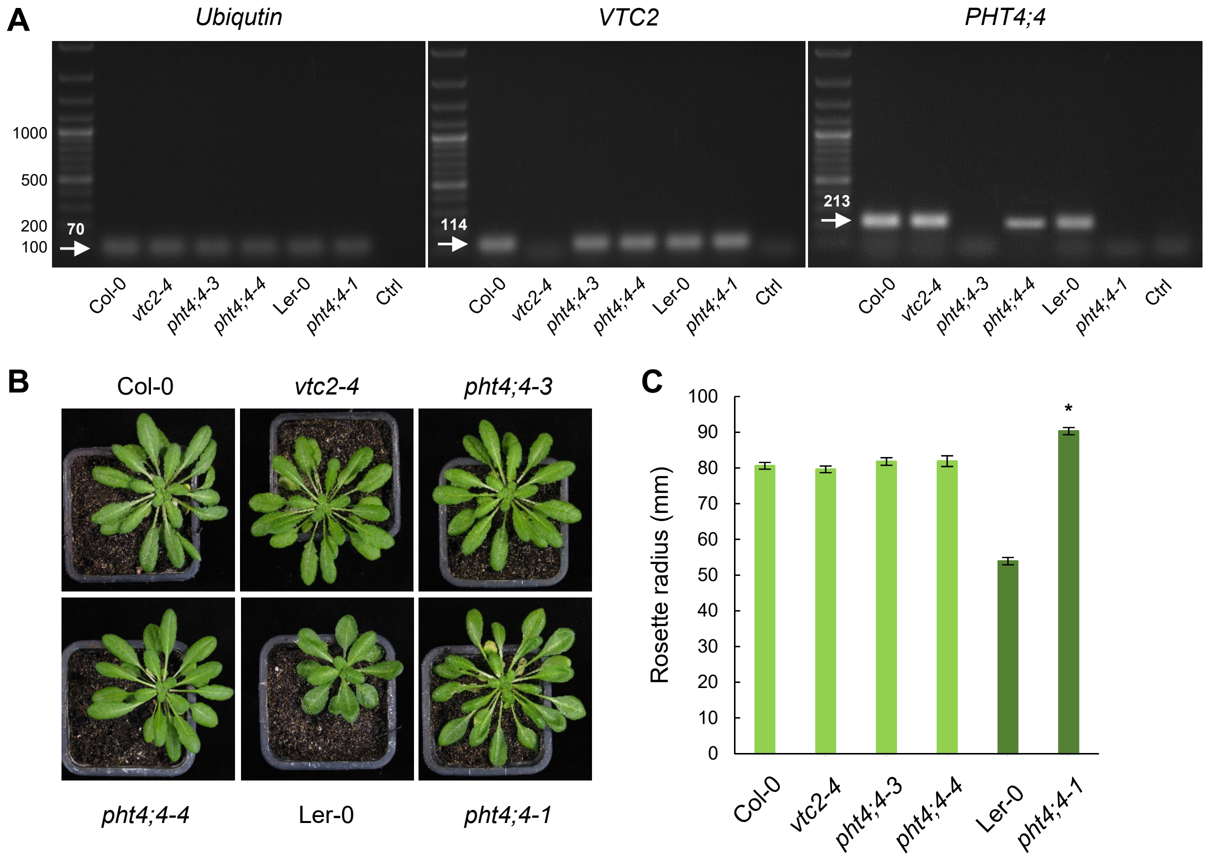

Supplementary Figure S1. Characterization of *PHT4;4* and *VTC2* mutants of Arabidopsis. **A)** RT-PCR performed using primers annealing downstream of the predicted insertion site in *PHT4;4* (primers P7 and P9), and *VTC2*. The expected sizes are marked with arrows. Ubiquitin was used as a reference gene, Ctrl stands for water-control. The number of RT-PCR cycles was 30. **B)** Phenotypes of eight-week-old *pht4;4-1*, *pht4;4-3*, *pht4;4-4*, and *vtc2-4* mutants and their respective WTs (Ler-0 and Col-0), grown at normal light (NL). **C)** Rosette sizes of eight-week-old plants grown at NL. The averages are based on six independent experiments, with 20 to 25 replicates in each and standard errors (±SE) are presented. Statistical significance levels between the mutants and their background strains were analyzed using Welch's unpaired t-test. The significance levels are presented as * ($p<0.1$). Primers are listed in Suppl. Table 2.

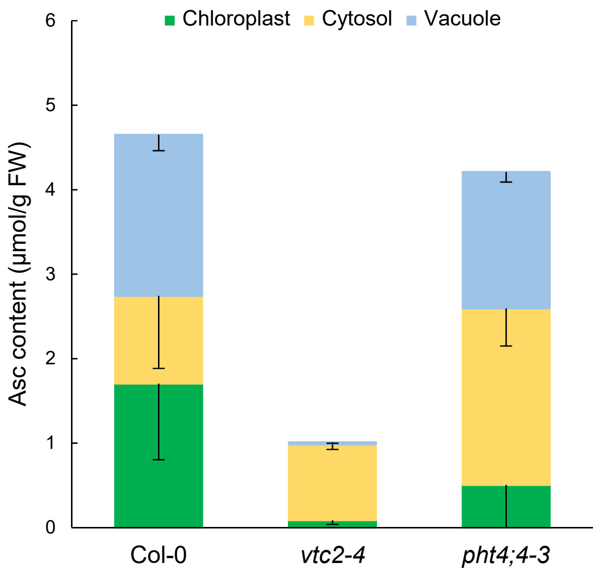

Supplementary Figure S2. The total ascorbate (Asc) content, distributed among the chloroplast, the cytosol and the vacuole, in the *vtc2-4* and *pht4;4-3* mutants and Col-0, assessed by non-aqueous fractionation. Data represent averages of three independent biological replicates with standard errors (±SE).

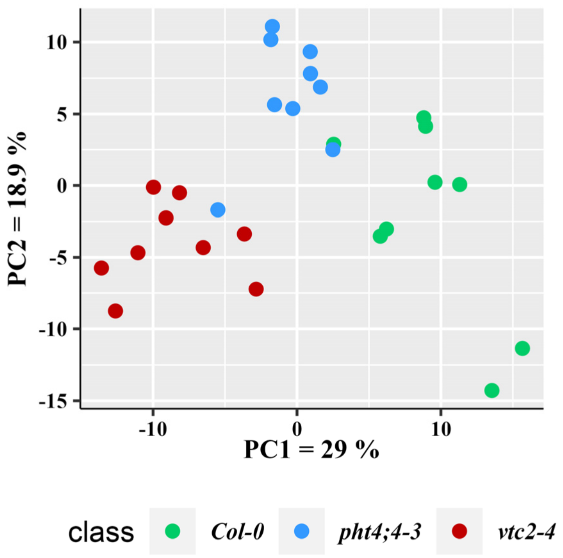

Supplementary Figure S3. Principal component analysis performed on Col-0, *vtc2-4*, and *pht4;4-3* mutant lines. The analysis was performed based on 244 metabolites, putatively annotated at MS1 and/or MS2 levels (levels "B" and "D", see Materials and Methods and Supplementary File 1).

A

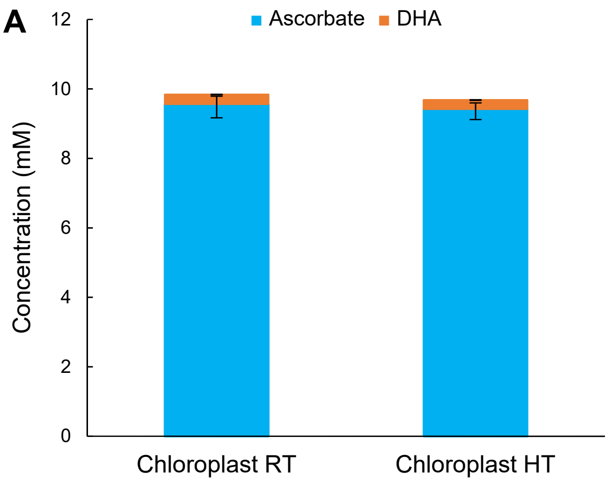

B

Sample	2-keto-gulonic acid (µM)	L-tartaric acid (µM)	Fraction of Asc degradation compounds (%)
RT + 10 mM Asc	21	4.1	0.26%
HT + 10 mM Asc	19.4	1.7	0.22%

Supplementary Figure S4. Concentration of ascorbate (Asc) and its degradation products in PISA-samples. 10 mM Asc was added to the samples, then incubated at room temperature (RT) or heat-treated at 55°C for 3 min (HT). **A)** HPLC analysis of Asc and dehydroascorbate (DHA) concentrations in the treated chloroplasts. The averages are based on three independent experiments, and standard errors (±SE) are presented. **B)** Concentration of the degradation products of Asc (2-keto-gulonic acid and L-tartaric acid), detected by mass spectrometry.

Supplementary Table S1. Metabolite changes specific to the *vtc2-4* mutant. The listed metabolites, identified at level "B" (see Materials and Methods), show significant ($p < 0.05$) difference after false discovery rate correction between *vtc2-4* and Col-0 lines and between *vtc2-4* and *pht4;4-3* and do not show significant difference ($p > 0.1$) between *pht4;4-3* and Col-0 genotypes. Equally probable alternative identifications are separated by "/".

**Metabolites identified at level "B"**
D-(+)-Proline
L-Threonine
Imidazoleacetic acid
L-Aspartic acid
L-Histidinol
DL-Lysine
L-Glutamic acid
Thiamine
Riboflavin
Succinic acid
Picolinic acid / nicotinic acid_1
4-Oxoproline
Glutaconic acid
Salicylic acid_1
Salicylic acid_3
D-(+)-Arabitol
Ascorbic acid
D-(-)-Fructose_2
L-Tyrosine
D-(-)-Mannitol
Galactonic acid / Gluconic acid

Supplementary Table S2. List of primer pairs used in this study.

Primer pairs	Sequences	Amplicon length (bp)	Specificity
**P1 + P3**	GGTTCCAACGAGTAGAAGATGA<br>ATGGAGATGCGTTCTGTAGATT	839	*pht4;4-1* gene specific
**P1 + P2**	GGTTCCAACGAGTAGAAGATGA<br>CCGTCCCGCAAGTTAAATATG	742	*pht4;4-1*; T-DNA specific
**P4 + P6**	CGGGCTACTATGCGTTTTGC<br>TAACCAGTAGCTGCAGTGCC	869	*pht4;4-3* gene specific
**P4 + P5**	CGGGCTACTATGCGTTTTGC<br>ATATTGACCATCATACTCATTGC	597	*pht4;4-3;* T-DNA specific
**P7 + P9**	TTCTCTCAGTCGGGTCTCTA<br>TGCGAACAAGTTCCAGACCA	213	*PHT4;4* (RT-PCR)
**P8 + P9**	GGCACTGCAGCTACTGGTTA<br>TGCGAACAAGTTCCAGACCA	108	*PHT4;4* (RT-qPCR)
***Ubiquitin* fw + rev**	CCCTCCACTTGGTCCTCAG<br>ATCGTCTTTCCCGTTAGGGTT	70	*Ubiquitin* (RT-PCR)
***VTC2* fw + rev**	TGACTGCTTGCCTCAAAGGA<br>GCAAAAGCACCCAAGCTGTT	114	*VTC2* (RT-PCR)
***PHT4;1* fw + rev**	TGTCACGAATGTCCGCAAGA<br>AAGCCATGCACAAAACAGCC	113	*PHT4;1* (RT-qPCR)
***PHT4;2* fw + rev**	TTATCGGAGGGGCATTGGTG<br>GCTGCCCAAGGAGTGAGTAG	100	*PHT4;2* (RT-qPCR)
***PHT4;3* fw + rev**	GCTTGGTTTAGTGCCGTTCC<br>ATGCTTGTACCACGCCGTAT	95	*PHT4;3* (RT-qPCR)
***PHT4;5* fw + rev**	TGCTGGATTTATGCCCGGTT<br>TGGCAATAAGGTCTGTCGCA	92	*PHT4;5* RT-qPCR
***PHT4;6* fw + rev**	AGAGTCGGGCTTTTGGTTGT<br>ACCCACTGAGCCAAAACTGT	92	*PHT4;6* RT-qPCR

***GAPDH* fw + rev**	TTGGTGACAACAGGTCAAGCA<br>AAACTTGTCGCTCAATGCAATC	62	*Glyceraldehyde-3-phosphate dehydrogenase* (RT-qPCR, ref. gene)
***ACT2* fw + rev**	CTTGCACCAAGCAGCATGAA<br>CCGATCCAGACACTGTACTTCCT<br>T	68	*Actin 2* (RT-qPCR, ref. gene)
***UBC21* fw + rev**	CTGCGACTCAGGGAATCTTCTA<br>A<br>TTGTGCCATTGAATTGAACCC	61	*Ubiquitin conjugating enzyme 21* (RT-qPCR, ref. gene)
