# Supplementary material for: Chloroplastic ascorbate modifies plant metabolism and may act as a metabolite signal regardless of oxidative stress
Source: Plant Physiol. 2024 Aug 6;196(2):1691–711. doi: 10.1093/plphys/kiae409 (PMC11444284; doi:10.1093/plphys/kiae409)
Supplement: kiae409_Supplementary_Data [file kiae409_supplementary_data.zip › PP2024RA00397DR1_Supplementary Texts 1 and 2.pdf]

Supplementary Text S1.

Detailed description of metabolite changes in the *vtc2-4* and *pht4;4-3* mutants and Col-0.

We explored how the metabolic responses are distributed across major compound classes, that is central and Asc metabolism, amino acids, and their derivatives, nucleotides and their derivatives, and secondary metabolites.

Regarding the central and Asc metabolites (Fig. 5A), 18 of the 31 metabolites detected were at significantly different levels in the *vtc2-4* mutant in comparison with Col-0 of which eight were significantly different in the *pht4;4-3* mutant as well.

Among the tricarboxylic acid (TCA) cycle intermediates, we find non-significant or minor decrease in the levels of fumaric acid, malic acid, and cis-acotinic acid, and a significantly increased level of 2-oxoglutaric acid in both mutants (Fig. 5A), which may indicate increased respiration (Araújo et al., 2014, Dastogeer et al., 2017). Succinic acid showed a minor decrease only in the *vtc2-4* mutant (Fig. 5A).

Regarding sugars, the levels of glucose 6-phosphate, and hexose phosphate remained in both mutants. The amounts of osmolyte sugars that are known to accumulate under oxidative stress conditions, such as raffinose, fructose, trehalose/sucrose, and mannitol (Baxter et al., 2007, Lehmann et al., 2009) remained or changed only mildly. Regarding organic acids, we found a significant increase in malonic acid content in both mutants, which is a compound in pyrimidine metabolism. Gluconic acid lactone and galactonic/gluconic acid contents also changed significantly, which indicates an alteration in the pentose phosphate pathway.

Carrying out the same analysis on the amino acids and their derivatives (Fig. 5B), revealed that the majority, i.e., 23 of the 38 metabolites detected were at significantly different levels in the *vtc2-4* mutant of which 12 were significantly different in the *pht4;4-3* mutant as well.

We observed that the amounts of proteinogenic amino acids increased mildly in the *vtc2-4* mutant, including tyrosine, lysine, aspartic acid, threonine, and glutamic acid. The slight accumulation of amino acids in the *vtc2-4* mutant may result from the activation of their biosynthetic pathways or from enhanced protein degradation (Ishikawa et al., 2009, Lehmann et al., 2009, Hildebrandt, 2018). These amino acids remained essentially unchanged in the *pht4;4-3* mutant. On the other hand, the levels of arginine and histidine increased in both mutants. Histidinol, a precursor of histidine decreased slightly in both mutants, whereas N-acetyl-DL-

glutamic acid, 3-methylhistidine and N-acetyl-L-arginine that are precursors or derivatives of arginine and histidine, increased.

The amounts of amino acids with antioxidant and osmolyte properties (proline, citrulline, spermidine) remained unchanged or decreased. The amount of the plant signaling molecule γ-aminobutyric acid (GABA, Xu et al., 2021) and its derivatives, 4-acetamidobutanoic acid and 4-guaidinobutyric acid increased in both mutants.

When analyzing the nucleotides and their derivatives (Fig. 5C), it could be seen that nine of the 18 metabolites detected were at significantly different levels in the *vtc2-4* mutant of which eight were significantly different in the *pht4;4-3* mutant as well, demonstrating that chloroplastic Asc had a large effect on nucleotide metabolism, too.

The amounts of uridine, uridine monophosphate, guanine, and adenosine 5'-monophosphate significantly increased in both mutants (Fig. 5C). We note that it was observed earlier that nucleotide metabolism and synthesis are rather diminished than intensified upon oxidative stress (Baxter et al., 2007). Instead, the increase in uridine and uridine monophosphate levels, and in malonic acid levels (Fig. 5A) indicates activation of the pyrimidine (uridine) salvage pathway that plays crucial roles in photoassimilate allocation and partitioning (Chen and Thelen 2011).

Pyridoxine (vitamin B6) and its vitamer derivatives, pyridoxal, and pyridoxamine have been shown to act as antioxidants and their levels increase strongly upon photooxidative stress (Chen and Xiong 2005; Havaux et al., 2009; Parra et al., 2018). Here, we observed a significant decrease in pyridoxine and pyridoxal levels in both mutants.

In purine catabolism, xanthine is oxidized to uric acid and then to allantoin, which process plays an important role in nitrogen-recycling and minimizing oxidative stress (Watanabe et al., 2010, Ma et al., 2016). Uric acid has been observed to decrease under oxidative stress conditions (Sipari et al., 2020); here we observed a significant decrease only in the *vtc2-4* mutant (Fig. 5C).

The level of UDP-N-acetylglucosamine slightly increased in both mutants (Fig. 5C), which is an essential amino sugar residue involved in N-glycan biosynthesis, protein glycosylation, and glycolipid biosynthesis (Decker and Kloczkowski, 2019).

Nicotinic acid levels increased in both mutants, and the level of nicotinamide was significantly higher in the *pht4;4-3* mutant than in Col-0 (Fig. 5C). Both nicotinic acid and nicotinamide are produced by the degradation of NAD and NADP in plant cells. From nicotinic acid trigonelline can be formed that may play a role in the detoxification of excess nicotinic acid and nicotinamide

(Ashihara et al., 2015). Since the level of trigonelline remained in both mutants (Fig. 5D), it is more likely that nicotinic acid and nicotinamide are re-utilised for the synthesis of pyridine nucleotides by salvage pathways. In plants, this cycle is also important in supplying nicotinic acid for the synthesis of pyridine alkaloids (Ashihara et al., 2015).

Finally, a comparison of secondary metabolites (Fig. 5D), revealed that 17 of the 29 metabolites detected were at significantly different levels in the *vtc2-4* mutant of which six of these being significantly different (either higher or lower) in the *pht4;4-3* mutant as well.

The levels of salicylic acid (tentatively identified as three separate components at level "B"), and the salicylic acid derivatives 3-furoic acid and gentisic acid remained in the *pht4;4-3* mutant, whereas the levels of some of these compounds changed significantly in the *vtc2-4* mutant (Fig. 5D).

Components of the phenylpropanoid pathway, including sinapinic acid (tentatively identified as three components), choline, and betaine mostly remained or diminished moderately (Fig. 5D). The level of anthranilic acid, an important early precursor of indole-3-acetic acid, which regulates the subcellular localization of auxin as well (Doyle et al., 2019), significantly decreased in both mutants (Fig. 5D).

The thiamine level slightly decreased in the *vtc2-4* and it remained in the *pht4;4-3* mutant (Fig. 5D). Thiamine functions as an important stress-response molecule that alleviate oxidative stress during different abiotic stress conditions (Tunc-Ozdemir et al., 2009; Rosado-Souza et al., 2020).

The levels of 2-aminoadipic acid and pipecolinic acid, which are both involved in lysine catabolism, have significantly increased in the *vtc2-4* mutant, whereas they only mildly increased in the *pht4;4-3* mutant (Fig. 5D), in agreement with the obtained lysine contents (Fig. 5B).

References:

Araújo WL, Martins AO, Fernie AR, Tohge T (2014) 2-oxoglutarate: linking TCA cycle function with amino acid, glucosinolate, flavonoid, alkaloid, and gibberellin biosynthesis. Front Plant Sci 5: 552

Ashihara H, Ludwig IA, Katahira, R. Yokota T, Fujimura T, Crozier A (2015) Trigonelline and related nicotinic acid metabolites: occurrence, biosynthesis, taxonomic considerations, and their roles in planta and in human health. Phytochem Rev 14: 765-798

Baxter CJ, Redestig H, Schauer N, Repsilber D, Patil KR, Nielsen J, Selbig J, Liu J, Fernie AR, Sweetlove LJ (2007) The metabolic response of heterotrophic Arabidopsis cells to oxidative stress. Plant Physiol 143: 312-325

Chen H, Xiong L (2005) Pyridoxine is required for post-embryonic root developmentand tolerance to osmotic and oxidative stresses. Plant J 44: 396-408

Chen, M, Thelen JJ (2011) Plastid uridine salvage activity is required for photoassimilate allocation and partitioning in Arabidopsis. Plant Cell 23: 2991-3006

Choi M (2014) MSstats: an R package for statistical analysis of quantitative mass spectrometry-based proteomic experiments. Bioinformatics 30: 2524-2526.

Dastogeer KMG, Li H, Sivasithamparam K, Jones M, Du X, Ren Y,Wylie SJ (2017) Metabolic responses of endophytic Nicotiana benthamiana plants experiencing water stress. Environ Exp Bot 143: 59-71

Decker D, Kleczkowski LA (2019) UDP-sugar producing pyrophosphorylases: Distinct and essential enzymes with overlapping substrate specificities, providing de novo precursors for glycosylation reactions. Front Plant Sci 9: 1822

Doyle SM, Rigal A, Grones P, Karady M, Barange DK, Majda M, Pařízková B, Karampelias M, Zwiewka M, Pěnčík A, Almqvist F, Ljung K, Novák O, Robert S (2019) A role for the auxin precursor anthranilic acid in root gravitropism via regulation of PIN-FORMED protein polarity and relocalisation in Arabidopsis. New Phytol 223: 1420-1432

Havaux M, Ksas B, Szewczyk A, Rumeau D, Franck F, Caffarri S, Triantaphylidès C (2009) Vitamin B6 deficient plants display increased sensitivity to high light and photo-oxidative stress. BMC Plant Biol 9: 130

Ishikawa T, Takahara K, Hirabayashi T, Matsumura H, Fujisawa S, Terauchi R, Uchimiya H, Kawai-Yamada M (2009) Metabolome analysis of response to oxidative stress in rice suspension cells overexpressing cell death suppressor Bax inhibitor-1. Plant Cell Physiol 51: 9-20

Joly D, Carpentier R (2011) Rapid isolation of intact chloroplasts from spinach leaves. In: Carpentier R (ed) Photosynthesis Research Protocols. Humana Press, Totowa, NJ, pp 321-325

Lehmann M, Schwarzländer M, Obata T, Sirikantaramas S, Burow M, Olsen CE, Tohge T, Fricker MD, Møller BL, Fernie AR, Sweetlove LJ, Laxa M (2009) The metabolic response of Arabidopsis roots to oxidative stress is distinct from that of heterotrophic cells in culture and

highlights a complex relationship between the levels of transcripts, metabolites, and flux. Mol Plant 2: 390-406

Ma X, Wang W, Bittner F, Schmidt N, Berkey R, Zhang L, King H, Zhang Y, Feng J, Wen Y, Tan L, Li Y, Zhang Q, Deng Z, Xiong X, Xiao S (2016) Dual and opposing roles of xanthine dehydrogenase in defense-associated reactive oxygen species metabolism in Arabidopsis. Plant Cell 28: 1108-1126

Parra M, Stahl S, Hellmann H (2018) Vitamin B6 and its role in cell metabolism and physiology. Cells 7: 84

Rosado-Souza L, Fernie AR, Aarabi F (2020) Ascorbate and thiamin: Metabolic modulators in plant acclimation responses. Plants 9: 101

Sipari N, Lihavainen J, Shapiguzov A, Kangasjärvi J, Keinänen M (2020) Primary metabolite responses to oxidative stress in early-senescing and praquat resistant Arabidopsis thaliana rcd1 (Radical-Induced Cell Death1). Front Plant Sci 11: 194

Tunc-Ozdemir M, Miller G, Song L, Kim J, Sodek A, Koussevitzky S, Misra AN, Mittler R, Shintani D (2009) Thiamin confers enhanced tolerance to oxidative stress in Arabidopsis. Plant Physiol 151: 421-432

Watanabe S, Nakagawa A, Izumi S, Shimada H, Sakamoto A (2010) RNA interference-mediated suppression of xanthine dehydrogenase reveals the role of purine metabolism in drought tolerance in Arabidopsis. FEBS Letters 584: 1181-1186

Xu B, Sai N, Gilliham M (2021) The emerging role of GABA as a transport regulator and physiological signal. Plant Physiol 187: 2005-2016

Supplementary Text S2.

Additional putative interactions between Asc and chloroplastic proteins indicated by the PISA assay

In addition to the interactions outlined in the main text, we found the following putative interactions between Asc and chloroplastic proteins:

We observed that the thermostability of plastidial dihydrolipoyl dehydrogenase 1 and 2 (LPD1 and LPD2) was diminished. LPD1 and LPD2 are the E3 subunits of the plastidial pyruvate decarboxylase complex (ptPDC) that is largely responsible for producing acetyl-coenzyme A and NADH for fatty acid biosynthesis in chloroplasts (Dörmann 2007). Fatty acids and fatty acid-derived complex lipids are essential components of cellular membranes, the precursors of cellular signaling molecules, such as jasmonic acid, and major energy reserves in storage tissues (Mooney et al., 2002).

The thermostability of 1-hydroxy-2-methyl-2-(E)-butenyl 4-diphosphate synthase (HDS or ISPG) was slightly increased by Asc. The methylerythritol phosphate (MEP) pathway is responsible for producing isoprenoids in the chloroplast. In addition, the MEP pathway intermediate MEcPP serves as a plastid-to-nucleus retrograde signal. ISPG activity affects MEcPP level (Wang et al., 2020), thereby it functions as a stress sensor and a coordinator of expression of targeted stress-responsive nuclear genes (Xiao et al., 2012, Wang et al., 2020). ISPG has an iron-sulfur cluster domain and receives electrons directly from the photosynthetic electron transport via ferredoxin or from ferredoxin-$NADP^+$ reductase (Seeman et al., 2006).

Asc increased the thermostability of phosphomethylpyrimidine synthase (THIC), an iron-sulfur cluster protein essential for thiamine biosynthesis thereby plant viability (Raschke et al., 2007, Rosado-Souza et al., 2020). The level of thiamine was diminished in the *vtc2-4* mutant.

A possible interaction was additionally observed between Ferritin 1 (FER1) and Asc at 5 mM Asc concentration (Fig. 6B), and in the presence of 10 mM Asc, the thermostability of FER3 and FER4 were increased, too (Suppl. File 1). These proteins are found in the chloroplast and may contain large amounts of Fe (Sági-Kazár et al., 2022). Ferritins are involved in protection against oxidative damage by sequestering Fe in an inert form to avoid Fenton reactions (Ravet et al., 2009). It has been shown that Asc may stimulate ferritin synthesis and it also increases the rate of iron

release from ferritin via reductive mechanisms (Lane and Richardson, 2014; Badu-Boateng and Naftalin, 2019).

We also found that Asc reduces the thermostability of NADPH-dependent thioredoxin reductases (NTR1, NTR2, also known as NTRA and NTRB, Fig. 6B). Thioredoxins play essential roles in redox regulation, and among many other roles, they are crucial in the redox regulation of the Calvin-Benson and the TCA cycle (reviewed by Nikkanen et al., 2017, Geigenberger et al., 2017). Thioredoxins are activated either by ferredoxin, using directly the reducing power of the photosynthetic electron transport chain, or by a NADPH-dependent thioredoxin system, functioning in all cellular compartments. NTRA and NTRB regulate the TCA cycle in the mitochondria but they are also found in the cytosol and probably in the nucleus, thereby they may have a pivotal role for the redox control plant metabolism (Daloso et al., 2015; Geigenberger et al., 2017). The observation that they were found in our chloroplast isolates raises the possibility that they are located in the chloroplast as well, although we cannot rule out the possibility of mitochondrial contamination, as such isolates cannot be perfectly devoid of contamination by other cellular compartments (Kueger et al., 2012).

Ascorbate increased the thermostability of CYP97C1 that is a ferredoxin-dependent non-heme P450-type carotenoid hydroxylase, which participates in the formation of a multienzyme complex that synergistically converts α-carotene to lutein in the chloroplast (Quinlan et al., 2012, McQuinn et al., 2015).

References:

Badu-Boateng C, Naftalin RJ (2019) Ascorbate and ferritin interactions: Consequences for iron release in vitro and in vivo and implications for inflammation. Free Radic Biol Med 133: 75-87

Brian P. Mooney, Jan A. Miernyk, and Douglas D. Randall (2002) The complex fate of α-ketoacids. Ann Rev Plant Biol 53: 357-375

Daloso DM, Müller K, Obata T, Florian A, Tohge T, Bottcher A, Riondet C, Bariat L, Carrari F, Nunes-Nesi A, Buchanan BB, Reichheld JP, Araújo WL, Fernie AR (2015) Thioredoxin, a master regulator of the tricarboxylic acid cycle in plant mitochondria. Proc Natl Acad Sci USA 17: E1392-1400

Dörmann P (2007) Lipid synthesis, metabolism and transport. In: The Structure and Function of Plastids (eds RR Wise and JK Hoober), pp. 335-353. Springer, Dordrecht, The Netherlands.

Geigenberger P, Thormählen I, Daloso DM, Fernie AR (2017) The unprecedented versatility of the plant thioredoxin system. Trends Plant Sci 22: 249-262

Kueger S, Steinhauser D, Willmitzer L, Giavalisco P (2012) High-resolution plant metabolomics: from mass spectral features to metabolites and from whole-cell analysis to subcellular metabolite distributions. Plant J 7: 39-50

Lane DJ, Richardson DR (2014) The active role of vitamin C in mammalian iron metabolism: much more than just enhanced iron absorption! Free Radic Biol Med 75: 69-83

McQuinn RP, Giovannoni JJ, Pogson BJ (2015) More than meets the eye: from carotenoid biosynthesis, to new insights into apocarotenoid signaling. Curr Opin Plant Biol 27: 172-179

Nikkanen L, Toivola J, Diaz MG, Rintamäki E (2017) Chloroplast thioredoxin systems: prospects for improving photosynthesis. Philos Trans R Soc Lond B Biol Sci 372: 20160474

Quinlan RF, Shumskaya M, Bradbury LM, Beltrán J, Ma C, Kennelly EJ, Wurtzel ET (2012) Synergistic interactions between carotene ring hydroxylases drive lutein formation in plant carotenoid biosynthesis. Plant Physiol 160: 204-214

Raschke M, Bürkle L, Müller N, Nunes-Nesi A, Fernie AR, Arigoni D, Amrhein N, Fitzpatrick TB (2007) Vitamin B1 biosynthesis in plants requires the essential iron-sulfur cluster protein, THIC. Proc Natl Acad Sci USA 104: 19637-19642

Ravet K, Touraine B, Boucherez J, Briat JF, Gaymard F, Cellier F (2009) Ferritins control interaction between iron homeostasis and oxidative stress in Arabidopsis. Plant J 57: 400-412

Rosado-Souza L, Fernie AR, Aarabi F (2020) Ascorbate and thiamin: Metabolic modulators in plant acclimation responses. Plants 9: 101

Sági-Kazár M, Solymosi K, Solti Á (2022) Iron in leaves: chemical forms, signalling, and in-cell distribution. J Exp Bot 73: 1717-1734

Seemann M, Tse Sum Bui B, Wolff M, Miginiac-Maslow M, Rohmer M (2006) Isoprenoid biosynthesis in plant chloroplasts via the MEP pathway: direct thylakoid/ferredoxin-dependent photoreduction of GcpE/IspG. FEBS Lett 580: 1547-1552

Wang JZ, Lei Y, Xiao Y, He X, Liang J, Jiang J, Dong S, Ke H, Leon P, Zerbe P, Xiao Y, Dehesh K (2020) Uncovering the functional residues of Arabidopsis isoprenoid biosynthesis enzyme HDS. Proc Natl Acad Sci USA 117: 355-361

Xiao Y, Savchenko T, Baidoo EE, Chehab WE, Hayden DM, Tolstikov V, Corwin JA, Kliebenstein DJ, Keasling JD, Dehesh K (2012) Retrograde signaling by the plastidial metabolite MEcPP regulatesexpression of nuclear stress-response genes. Cell 149: 1525-1535
